# Sequencing genes in silico using single nucleotide polymorphisms

**DOI:** 10.1186/1471-2156-13-6

**Published:** 2012-01-30

**Authors:** Xinyi Cindy Zhang, Bo Zhang, Shuying Sue Li, Xin Huang, John A Hansen, Lue Ping Zhao

**Affiliations:** 1 Division of Public Health Sciences, Fred Hutchinson Cancer Research Center, Seattle, WA, 98109, USA; 2 Division of Clinical Research, Fred Hutchinson Cancer Research Center, Seattle, WA, 98109, USA; 3 School of Medicine, University of Washington, Seattle, WA, 98195, USA

**Keywords:** In silico, SNPs, 1000 Genomes Project, multi-allelic gene, imputation

## Abstract

**Background:**

The advent of high throughput sequencing technology has enabled the 1000 Genomes Project Pilot 3 to generate complete sequence data for more than 906 genes and 8,140 exons representing 697 subjects. The 1000 Genomes database provides a critical opportunity for further interpreting disease associations with single nucleotide polymorphisms (SNPs) discovered from genetic association studies. Currently, direct sequencing of candidate genes or regions on a large number of subjects remains both cost- and time-prohibitive.

**Results:**

To accelerate the translation from discovery to functional studies, we propose an in silico gene sequencing method (ISS), which predicts phased sequences of intragenic regions, using SNPs. The key underlying idea of our method is to infer diploid sequences (a pair of phased sequences/alleles) at every functional locus utilizing the deep sequencing data from the 1000 Genomes Project and SNP data from the HapMap Project, and to build prediction models using flanking SNPs. Using this method, we have developed a database of prediction models for 611 known genes. Sequence prediction accuracy for these genes is 96.26% on average (ranges 79%-100%). This database of prediction models can be enhanced and scaled up to include new genes as the 1000 Genomes Project sequences additional genes on additional individuals. Applying our predictive model for the KCNJ11 gene to the Wellcome Trust Case Control Consortium (WTCCC) Type 2 diabetes cohort, we demonstrate how the prediction of phased sequences inferred from GWAS SNP genotype data can be used to facilitate interpretation and identify a probable functional mechanism such as protein changes.

**Conclusions:**

Prior to the general availability of routine sequencing of all subjects, the ISS method proposed here provides a time- and cost-effective approach to broadening the characterization of disease associated SNPs and regions, and facilitating the prioritization of candidate genes for more detailed functional and mechanistic studies.

## Background

The recent advent of SNP array based high throughput genotyping technologies has stimulated a wave of genetic association studies, including genome-wide association studies (GWAS) [1], and has led to a number of discovered and validated associations, which are fully catalogued on the website http://www.genome.gov/gwastudies/. While many of the disease associations were found with single nucleotide polymorphisms (SNPs) in non-coding regions, some are found with SNPs within or nearby known functional genes. To move beyond SNP associations, a common approach is to sequence the candidate gene region in affected subjects and fully define the entire variation in the region. With this, one could study the disease association with the gene via natural "genetic alleles" before launching more detailed mechanistic studies [2]. Despite the recent advances in high-throughput sequencing technologies (e.g., Solexa of Illumina http://www.illumina.com/, 454 Life Sciences http://www.454.com/, Solid of Applied Biosystem http://www.appliedbiosystems.com), sequencing targeted genes or regions for a large number of samples remains cost-prohibitive.

In this study, we describe an in silico sequencing (ISS) method to more fully define the sequence variations in candidate gene regions. Recently, the 1000 Genomes Project http://www.1000genomes.org has completed its initial phases, including a pilot project which has sequenced 8,140 target exons from 906 selected gene regions in 697 subjects with an average depth of 50X. With this high quality sequence data, we were able to deduce a database of multi-allelic gene polymorphisms for a majority of the selected gene regions. Based upon the linkage-disequilibrium between the inferred gene alleles and the SNP genotype data of these subjects, we constructed models for predicting a pair phased sequences of these genes using selected flanking SNPs. Such prediction models can be used to sequence candidate genes in silico with minimal genotyping requirement.

This development is an extension to our recently developed method [3], which predicts Human Leukocyte antigen (HLA) gene alleles. The key idea is to model gene alleles as multi-allelic polymorphisms, and to build a likelihood framework of all gene alleles and genotypes of flanking SNPs for the sample set. Previously, we have demonstrated success in predicting HLA-A, -B, -C, -DR and -DQ genes in high resolution with accuracy 95, 93, 97, 79, and 83% respectively. Of course, HLA genes are quite special, since 1) these genes are among the most polymorphic genes known to date, 2) there is extensive linkage disequilibrium (LD) in the region, 3) large sets of samples have been directly sequenced for their HLA gene alleles with resolved phasing information in hematopoietic stem cell transplantation (HSCT), or studies of autoimmune diseases, such as Type 1 diabetes. In this study, we show that it is possible to build comparable prediction models with satisfactory accuracies for other genes by treating diploid sequences in the gene region as large multi-allelic polymorphisms.

This method of predicting polymorphic genetic variants is closely related to the commonly used imputation methods. Typically, imputation methodologies such as Impute [4,5], Mach [6], and BEAGLE [7], are designed to impute untyped SNPs for GWAS on a particular commercial SNP arrays (e.g. Affymetrix 6.0 or Illumina 1.2 M array), based upon a reference panel of a more dense set of SNPs (e.g. the HapMap SNPs). While utilizing underlying LD structure, these imputation methods typically aim to produce "imputed genotypes" for single nucleotide variants. In contrast, we have tuned our approach to predict (or impute) a pair of phased gene alleles. The primary reason for our focus is that gene alleles, typically associated with functional sequences in the coding gene region, can be readily converted to amino acid sequences for further interpretation. In addition, one can naturally incorporate structural variations into allele polymorphisms, due to the modeling of the entire sequence region.

In the remainder of the paper, we first describe the proposed methods then summarize the properties of genes deep sequenced in the 1000 Genomes Project, and evaluate the performance of our gene prediction models in the Results Section. Then we present an application of our approach to an existing GWAS data gathered from the WTCCC. We conclude this paper with discussions on the strengths and limitations of the proposed method.

## Methods

### Deducing Diploid Sequences

In order to build prediction models for gene diploid sequences, one of the first steps is to deduce the phase information of the gene sequences for the training dataset, which are the samples sequenced in Pilot 3 of the 1000 Genomes Project here. Utilizing all SNP calls within each gene, we deduced diploid sequences for every subject. This is equivalent to phasing multiple SNPs, in the absence of any structural variants as in the current case. Several methods have been developed for phasing multiple SNPs [8-12]. The empirical method for estimating haplotype frequencies and for inferring haplotypes as described in [8] and implemented in HPlus http://qge.fhcrc.org/hplus/ was used here to efficiently phase the genotypes even when the number of SNPs was large.

### Predicting Multi-Allelic Genes

With these phased gene diploid sequences, we expanded the previously developed methodology [3] to predict alleles of genes sequenced in the 1000 Genomes Project Pilot3. The key idea is to model gene diploid sequences as multi-allelic polymorphisms, and to build a likelihood framework of all gene alleles and genotypes of flanking SNPs for the sample set. That is, given a random sample of N subjects, we denote the genotype of a gene by $h_{i}={ḣ}_{i}{ḧ}_{i}$, where ${ḣ}_{i}$ and ${ḧ}_{i}$ are the two alleles of the gene for the i^th ^subject. The genotypes of the q SNPs flanking the gene region is denoted by *g_i _*= (*g_i_*_1_, *g_i_*_2_, ..., *g_iq_*), where g_ij _is the SNP genotype at the *j*^th ^locus for the i^th ^subject. If the phase at each locus is known, then the genotypes of the q SNPs can be represented by $g_{i}=\left({Ġ}_{i},{\ddot{G}}_{i}\right)$, where ${Ġ}_{i}$ and ${\ddot{G}}_{i}$ are the SNP haplotypes. The gene-SNP haplotypes *hG *is assumed to follow a multinomial distribution expressed as $f\left({ḣ}_{i}{Ġ}_{i}\right)=\prod_{\underline{hG}\in \Theta }\text{Pr}{\left(\underline{hG}\right)}^{I\left({ḣ}_{i}{Ġ}_{i}=\underline{hG}\right)}$, where $\Theta =\Omega \left(h_{i},g_{i}\right)$ includes all haplotype pairs (*hG*) that are consistent with the observed genotypes (h_i_, g_i_). The frequencies of gene-SNP haplotypes Pr(*hG*) are estimated by maximizing the log likelihood function

$$l=\overset{N}{\underset{i=1}{\sum }}\text{log}f\left(h_{i},g_{i}\right)=\overset{N}{\underset{i=1}{\sum }}\text{log}\left(\underset{\left({ḣ}_{i}{Ġ}_{i},{ḧ}_{i}{\ddot{G}}_{i}\right)\in \Omega \left(h_{i},g_{i}\right)}{\sum }f\left({ḣ}_{i}{Ġ}_{i},{ḧ}_{i}{\ddot{G}}_{i}\right)\right),$$

which leads to

$$l=\overset{N}{\underset{i=1}{\sum }}\text{log}\underset{\left({ḣ}_{i}{Ġ}_{i},{ḧ}_{i}{\ddot{G}}_{i}\right)\in \Omega \left(h_{i},g_{i}\right)}{\sum }f\left({ḣ}_{i}{Ġ}_{i}\right)f\left({ḧ}_{i}{\ddot{G}}_{i}\right)$$

under the Hardy-Weinberg equilibrium (HWE). The detailed estimation procedure was described in [8]. The gene alleles are then predicted following the Bayesian rule as:

$$f\left(h|g\right)=\frac{f\left(h,g\right)}{\underset{H=ḢḦ}{\sum }f\left(H,g\right)}=\frac{\underset{\left(ḣĠ,ḧ\ddot{G}\right)\in \Omega \left(h,g\right)}{\sum }\underset{\underline{hG}}{\prod }\text{Pr}{\left(\underline{hG}\right)}^{I\left(ḣĠ=\underline{hG}\right)+I\left(ḧ\ddot{G}=\underline{hG}\right)}}{\underset{ḢḦ}{\sum }\underset{\left(ḢĠ,Ḧ\ddot{G}\right)\in \Omega \left(H,g\right)}{\sum }\underset{\underline{hG}}{\prod }\text{Pr}{\left(\underline{hG}\right)}^{I\left(ḢĠ=\underline{hG}\right)+I\left(Ḧ\ddot{G}=\underline{hG}\right)}}.$$

We build a procedure to systematically select the most informative SNPs using a forward-and-backward scheme, which starts with the SNPs within a gene (if available) and gradually extends to flanking regions of each gene. The SNP selection process was evaluated by an objective function, which is the negative log-likelihood of the gene allele given SNP genotypes penalized by the number of additional parameters to be estimated (Akaike information criterion (AIC) [13]) as follows:

$$Q=-\overset{N}{\underset{i=1}{\sum }}\text{log}f\left(h_{i}|g_{i}\right)+\left(m-k\right),$$

where the second term equals the difference of the number of gene-SNP haplotypes (m) and the number of gene alleles (k).

The search region was empirically chosen to maximize the prediction accuracies while retaining parsimony. We have chosen 150 kb flanking region to predict HLA genes in our previous work [3]. However, when building prediction models to accommodate multiple genes, we had to choose the searching boundary generally applicable to all genes. In this study, we evaluated the performance of flanking regions of size 0 to 500 kb with a step size of 50 kb for each gene. In order to compare the relationship between objective function and size of flanking region among all selected genes, we rescale the objective function values (*O_g_*) for gene *g*, denoted as Ô_g_, by (*O_g_*-min(*O_g_*))/(max(*O_g_*)-min(*O_g_*)), where the minimum and maximum of *O_g _*is taken across all sizes of flanking region. By definition, these rescaled objective function values are within range 0 [1]. The goal was to choose a size of flanking region such that Ô_g _reaches its minimum for a majority of the genes. In addition, to accommodate genes with different complexity level, we have incorporated several modifications (see Additional file 1) to ensure computational efficiency and feasibility.

#### Prediction accuracies

As part of the model validation, we applied prediction models built from the training set, to predict diploid sequences on another dataset, and to compare the predictions with the actual diploid sequences. For this comparison, we used the following accuracy measurement. Suppose that $D_{ip}={ḣ}_{ip}|{ḧ}_{ip}$ and $D_{io}={ḣ}_{io}|{ḧ}_{io}$denote the predicted and observed diploid sequences for the *i*th subject respectively. Then the prediction accuracy, over all subjects in the validation set, was measured by the percentage of correctly predicted diploid sequences, i.e.

$$Accuracy=\frac{1}{2N}\overset{N}{\underset{i=1}{\sum }}n_{i},$$

where *N *is the number of subjects and

$$n_{i}=\max\left[I\left({ḣ}_{ip}={ḣ}_{io}\right)+I\left({ḧ}_{ip}={ḧ}_{io}\right),I\left({ḣ}_{ip}={ḧ}_{io}\right)+I\left({ḧ}_{ip}={ḣ}_{io}\right)\right]$$

represents the number of correctly predicted diploid sequences for the ith subject.

#### Measuring genetic polymorphism

Typical genes under consideration are polymorphic, i.e., with multiple alleles. To measure such polymorphisms, we propose to use the Shannon entropy, which is commonly used to measure variability of discrete variable [14]. Consider a gene with *n *alleles, with allelic frequencies (*f*_1_, *f*_2_, ..., *f_n_*). The entropy of this gene was defined as

$$E\left(G\right)=-\overset{n}{\underset{i=1}{\sum }}f_{i}\log\left(f_{i}\right),$$

where the summation is over all possible alleles. Entropy with value of 0 means that the gene is not polymorphic. The maximum value of entropy equals to *log(n)*. In general, the larger the entropy is, the more polymorphic is the gene.

### Analysis Pipeline

Here we describe a general analysis strategy for building prediction models for diploid sequences of genes sequenced in the 1000 Genomes Project using SNPs. The resulting prediction models can be easily applied to sequence candidate genes in silico and facilitate the interpretation of association discoveries. To build reliable prediction models based on a relatively small number of samples from each population (Table 1), one can combine samples from multiple ethnicities [15]. To separate the training and validation processes, we randomly selected half of the samples from each of the seven selected study populations into the training set and designated the remaining samples to the validation set.

**Table 1:** Samples genotyped in the 1000 Genomes Project pilot3 and the HapMap3 Project

Populations\Projects		1000 Genomes	HapMap3*	Both projects
		Project pilot3^#^		
European	CEU	89	165	83
	
ancestry	TSI	66	102	61

East Asian	CHB	109	137	89
	
ancestry	CHD	107	109	90
	
	JPT	105	113	89

West African	YRI	111	203	104
	
ancestry	LWK	108	110	98

# The 1000 Genomes project pilot3 sequenced two trio samples from CEU and YRI. The children in these families were excluded from this analysis.

* Only samples that were in the same 7 populations of the 1000 Genomes project were listed here. The HapMap3 project also genotyped samples from ASW (African ancestry in Southwest USA), GIH (Gujarati Indians in Houston, Texas), MEX (Mexican ancestry in Los Angeles, California), and MKK (Maasai in Kinyawa, Kenya).

Given the large amount of sequencing data, the typical first step is to align short-read sequences to the reference genome, and then to make variant callings. In this project, investigators from the 1000 Genomes Project have already performed such analysis, producing all possible SNP genotype calls in each study population.

The second step is to clean the data. For each gene, we filtered out samples that have at least one SNP missing in the gene. Genes that have more than 50% of samples filtered or have only one SNP were excluded from analysis. In addition, samples that were filtered in more than 60% of genes were excluded.

The third step is to deduce diploid sequences by phasing genotypes of all SNPs within every gene for every subject. To maintain phasing efficiency and at the same time retain the independence of the training and validation sets, we estimated the frequency of alleles of each gene using the entire set of samples, and then predicted the phases of gene alleles for samples in the training and validation set separately. Each distinct gene allele was then assigned a label as GeneName*Allele, where Allele was labeled by the numerical number 1, 2,..., in the order of the frequency of corresponding gene allele. The diploid sequences were called for each subject, if the corresponding posterior probability was maximum and also exceeded a pre-assigned threshold. Otherwise, the subject's sequence pair was deemed as missing. To ensure the quality of prediction models, a stringent threshold of probability > 0.95 was applied and only genes that had less than 10% samples with missing diploid sequences in both training and validation sets were selected for further model building.

The fourth step is to build prediction models for each gene using our algorithm of predicting multi-allelic genes. The prediction models built based on diploid sequences and SNP genotype data of samples in the training set were then applied to the training set itself. The prediction accuracy on the training set was thus evaluated by comparing the predicted diploid sequences with the observed ones. To control the quality of prediction accuracy, we used call threshold (CT) of CT = 0, 0.5 and 0.9, i.e., one would make a prediction only if the posterior probability exceeds CT.

The last step is to validate these prediction models. By applying the prediction models built in the above step to the validation set, we computed the prediction accuracy using CT = 0, 0.5 and 0.9, which would reflect "true" prediction accuracies in practice.

## Results

### Deeply Sequenced Genes

**Figure 1:**
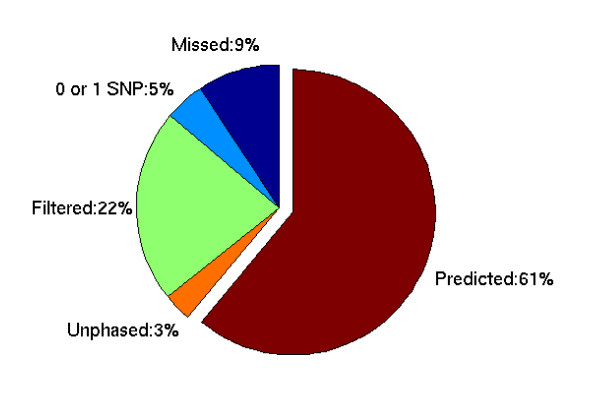
**Categories of genes targeted in the 1000 Genomes Project Pilot3**. Of the 1,000 CCDS genes targeted, 93 genes (Missed) were not targeted successfully in at least one genotyping center, 45 genes (0 or 1 SNP) have only zero to 1 SNP called, 219 genes (Filtered) were filtered out (≥ 50% of the samples have missing value in at least one SNP within the gene), 32 genes (Unphased) were not phased properly (≥ 10% of the samples have phasing probability < 0.95 in either training or validation set), the remaining 611 genes (Predicted) were selected for analysis in this project.

All variants from Pilot 3 of the 1000 Genomes Project have been identified and organized in a variant file [16]. Briefly, a total of 1,000 CCDS (Consensus Coding Sequences: http://www.ncbi.nlm.nih.gov/CCDS/CcdsBrowse.cgi) genes were considered, and were represented by 8,496 exon targets. Of these genes, there were 907 autosomal genes with a total of 8,174 consensus target exons successfully captured and sequenced at multiple centers for 695 independent individuals from seven populations (Table 1). Except for 21 insertions/deletions, nearly all polymorphisms called in these exons are SNPs. Thus, all sequence variations were effectively represented by SNPs. By linking SNPs with their specific genes, we found that 862 of 907 genes (95%) have two or more SNPs. For each gene, we filtered out samples that have one or more SNPs missing in the gene. Genes that have more than 50% of samples filtered or have only one SNP were excluded from analysis. In addition, samples that were filtered in more than 60% of genes were excluded. There are 643 genes and 684 samples remaining for further analysis (see Figure 1 for the breakdown).

### Properties of Selected Genes

**Figure 2:**
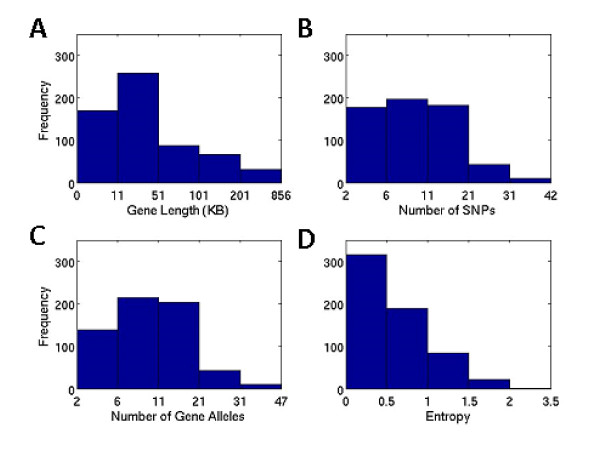
**Histograms of the characteristics of genes (N = 611) selected in this project**. Note that the interval length of each group is not evenly distributed.

Utilizing all SNPs in the gene, we successfully deduced diploid sequences for 611 of the 643 genes (See Methods Section for further information). Of these genes selected for the following modeling exercise, 84% are less than 100 KB, while the remaining are larger, with the largest being 856 KB in size (Figure 2a). The number of SNPs in each gene ranges from 2 to as many as 42 (Figure 2b). The majority of the genes have between 6 and 21 identified alleles, while 55 of the genes (9%) have at least 21 to 41 alleles (Figure 2c). The broad range of polymorphisms among the 611 genes is further illustrated by the distribution of gene entropies (defined in Methods Section)(Figure 2d). See Additional file 2 for a more detailed summary of gene properties.

### Training Prediction models

Of the individuals sequenced in Pilot 3, 614 samples from multiple populations have been genotyped in HapMap 3 using the Affymetrix Human SNP array 6.0 and the Illumina Human 1M-single BeadChip. We have recently shown that prediction models built using a training set that includes multi-ethnic populations are reliable and perform well for the subpopulations [15]. Thereby we combined samples from different ethnicities in this model building process.

**Figure 3:**
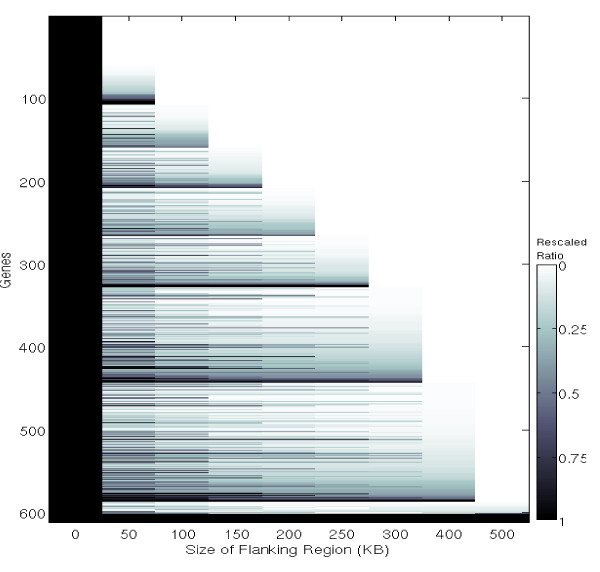
**Plot of rescaled objective function value versus the flanking region size**. The prediction models for the set of 611 genes selected were built using nine different flanking region sizes, shown on x axis. The y axis shows the list of genes (name not shown). For each gene g, the objective function value *O_g _*is rescaled as (*O_g_*-min(*O_g_*))/(max(*O_g_*)-min(*O_g_*)). The change of this rescaled ratio for different flanking sizes is plotted on row g using the color lightness as shown in the color bar.

Using the training set, we built prediction models for all 611 genes and plotted the rescaled objective function value Ô_g _(See Methods Section for further details) for each gene at each flanking region size, quantified by the color intensity as shown in the color bar. As illustrated in Figure 3, Ô_g _generally decreases as the flanking size increases. While Ô_g _for some genes continues to decrease as SNPs up to 500 kb away from the gene were selected, it reaches its minimum for about half of the genes at flanking size 250 kb. To balance between prediction accuracy and the possible over-fitting problem, we chose to use 250 kb as the size of flanking region for our further analysis.

**Figure 4:**
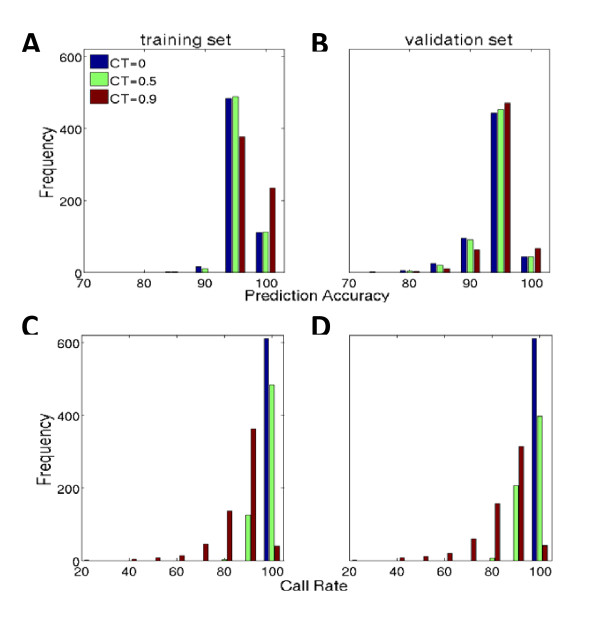
**Prediction accuracy (%) and Call rate (%) of prediction models built using flanking region size 250 K in the training set (N = 342) and the validation set (N = 342)**.

Upon the completion of the model building, we predicted diploid sequences for the training set using prediction models, and compared prediction results with observed diploid sequences. Using call threshold (CT) as 0, 594 out of 611 genes have predictive accuracies averaging 98.26% (range, 95% to 100%) (Figure 4a). Increasing CT to 0.5 and 0.9 improves prediction accuracies to 98.34% and 99.23% respectively, at the expense of reducing call rates (Figure 4c). Balancing between prediction accuracies and call rates, this exercise supports the use of CT = 0, which is taken as default hereafter.

**Figure 5:**
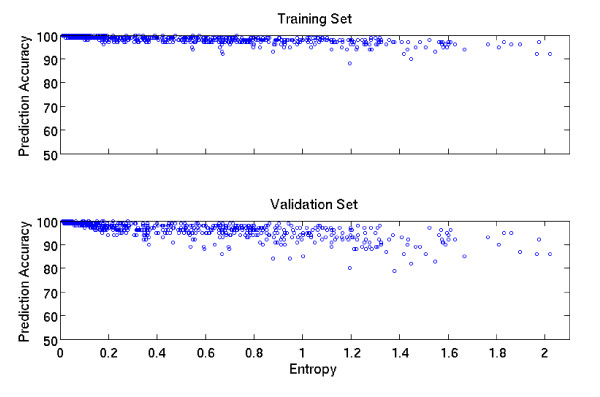
**Relationship of the accuracy of prediction models with gene entropy in the training set and the validation set at CT = 0**.

Prediction accuracies were examined in the relation to entropy (Figure 5). In the training set (top panel), prediction accuracies are largely above 0.95. However, there is a tendency for the prediction accuracy to decline as the entropy increases.

### Validating Prediction models

The prediction models built from the training set were validated by predicting sequence variants for each of the 611 genes in the independent validation sample set. The accuracy of the prediction was evaluated by comparing between the predicted and observed diploid sequences. The distributions of prediction accuracy with CT = 0, 0.5 and 0.9 are illustrated in Figure 4b, and the distributions of the call rates corresponding to CT = 0, 0.5 and 0.9 are illustrated in Figure 4d. As expected, the call rates decline as CT increases from 0 to 0.9. Interestingly, the accuracies with any CT largely peaked at the interval [95%, 100%), among about 500 out of 611 genes. About 50 genes have accuracies approaching to 100% and slightly more than 50 genes have accuracies around 90%. The relationship between the prediction accuracy and the variability of gene is similar to that shown in the training set (Figure 5). For more details, see Additional file 3.

### Final Model

The training and validation exercises described above have shown that the proposed method of in silico sequencing candidate genes is valid, scalable, and has satisfactory prediction accuracies for a broad sampling of genes. To make the gene prediction models more robust, we combined the training and validation sets to build the final prediction models for all 611 genes using the procedure described above. The prediction models included the number of SNPs ranged from 1 to 38. Those models and the SNPs selected are available on our website http://qge.fhcrc.org/ISS/.

### KCNJ11: A Type 2 Diabetes Associated Gene

To illustrate the utility of the prediction model in silico gene sequencing, we analyzed the Type 2 diabetes (T2D) GWAS data generated by WTCCC [17]. The WTCCC T2D study includes 2,000 cases and 3,000 controls. Genotyping was done using the Human GeneChip 500 K Mapping Array (Affy500K). WTCCC investigators have successfully replicated 13 disease associated variants which include the SNPs located in KCNJ11 and PPARG gene. KCNJ11 gene has been deeply sequenced for its exon region in the 1000 Genomes Project Pilot 3. Thus, using KCNJ11 as an example, we illustrate how one would use our prediction models to sequence KCNJ11 gene in silico, and to advance the validation/functional study of the discovered SNP association before actually sequencing the gene in the wet lab.

**Figure 6:**
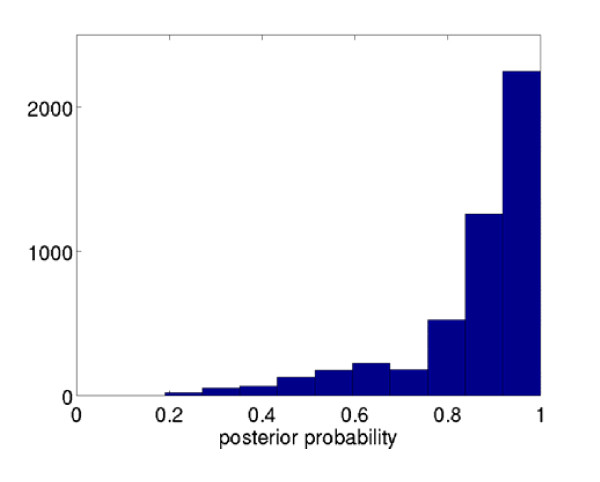
**Distribution of predictive probabilities (the highest posterior probability among predicted KCNJ11 alleles for every subject) in the WTCCC data**.

The predictive model was built for KCNJ11 gene following the above modeling process, using the available SNPs from HapMap 3. Since not all selected SNPs were genotyped in WTCCC, we employed IMPUTE v2 to impute the additional SNPs, using default parameters. With both the imputed and genotyped SNPs from WTCCC, we sequenced the candidate gene KCNJ11 in silico based upon the predictive model. The distribution of predictive probabilities is illustrated Figure 6. It shows that satisfactory prediction confidences (p > 0.8) can be achieved for > 77% of samples.

Nine gene alleles were predicted to be in both case and control sets. Using the most frequent allele (KCNJ11*3) as the reference, we performed a multi-allelic association test using HPlus [8] for the five common gene alleles with frequency more than 0.01 in both cases and controls. As shown in Table 2, KCNJ11*1 and KCNJ11*2 were significantly associated with T2D with p-value 0.022 and 0.036 respectively. Both of these significant gene alleles differed from the reference allele only by two SNPs: rs5215 and rs5219. This indicated the possible functionality of these SNPs. Indeed, rs5215 was the SNP reported in the WTCCC study, and rs5219 has been independently reported the association with T2D [18]. The two SNPs were in high LD with correlation r^2 ^= 0.9. However, rs5219 was not directly analyzed in the WTCCC since it was not genotyped on the Affy500K array. In addition, KCNJ11*1 also had the variant allele of synonymous SNP rs5218. This SNP might not be as critical as the other two SNPs, since the T2D association was independent of its alleles. The other common gene allele KCNJ11*5 differed from the reference allele only at rs1800467 and was not significantly different from KCNJ11*3 with regard to T2D association.

**Table 2:** Results of the multi-allelic association of the KCNJ11 gene alleles with T2D.

Haplotype		Frequency							
								
Common	Variations*	Control	Case	Coef	SE	OR	95% CI	Z-score	p-value
KCNJ11*3	GC**C**G**G**G**G**C**T**	0.336	0.364			1			
KCNJ11*2	GC**T**G**G**G**G**C**C**	0.320	0.309	-0.11	0.05	0.9	(0.81,0.99)	-2.09	0.036
KCNJ11*1	GC**T**G**G**G**A**C**C**	0.317	0.301	-0.12	0.05	0.89	(0.8, 0.98)	-2.29	0.022
KCNJ11*5	GC**C**G**C**G**G**C**T**	0.015	0.018	0.1	0.16	1.1	(0.8, 1.52)	0.59	0.558
**Uncommon**									
KCNJ11*8	GT**T**G**G**G**G**C**C**	0.008	0.006						
KCNJ11*4	GC**T**G**G**C**G**C**C**	0.0014	0.0005						
KCNJ11*6	GC**C**T**G**G**G**C**T**	0.00078	0.001						
KCNJ11*7	CC**T**G**G**G**G**C**C**	0.001	0.0003						
KCNJ11*13	GC**TGG**G**G**A**C**	0.0005	0						

In WTCCC1 cohort, which have 1924 T2D cases and 2938 controls (NBS and 58BC), a total of 9 gene alleles were predicted with variations at 9 SNP locations (rs41282930, rs8175351, **rs5215**, rs1800854, r**s1800467**, rs5216, **rs5218**, rs0^#^, **rs5219**). The 4 common gene alleles (freq > 0.01) were analyzed in a multi-allelic association in this case-control study.

*The SNP variations among the common haplotypes (freq > 0.01) are highlighted in bold.

#This SNP is located at chr11:17366025 (NCBI build 36) and has not been assigned an rs number.

To investigate the functional basis of these variants, we examined the amino acid sequence variants for KCNJ11 gene. Utilizing the reference genome from NCBI http://www.ncbi.nlm.nih.gov/, we converted the diploid sequences of KCNJ11 gene to their amino acid sequences. Due to redundant codon usage, the number of amino sequence variants decreased from nine to five. With the reduced number of variants, we anticipated an increase in statistical power. Taking the most frequent aa*5 as reference, there were two common amino acid sequence variants, i.e. aa*8 and aa*9, with frequency > 0.01 (Table 3). Both sequences consisted of two amino acid variants, namely E23K and I337V, which corresponded to exactly the two SNP variants rs5219 and rs5215 found in the significant genomic sequence variants. The multi-allele association analysis showed that only aa*8 was significantly associated with T2D and the p-value is 0.003. This association was comparable to the significant association of genomic sequence variants KCNJ11*3 comparing to KCNJ11*1 and KCNJ11*2. However, due to the reduction of the total number of sequence variants tested for associations, we observed more significant p-values, reflecting the increase in statistical testing power. In addition, the association analysis of amino acid sequence variants also indicates a potential functional interaction of amino acid variants L270V with E23K and I337V, since having an additional variant L270V compared to aa*8 leads to the insignificant association of sequence variant aa*9 with T2D. However, the frequency of aa*9 is rather low at 44 copies in the control and 34 copies in the case.

**Table 3:** Results of the multi-allelic association of the KCNJ11 amino acid gene alleles with T2D.

Haplotype		Frequency							
								
**Common**	Variations*	Control	Case	Coef	SE	OR	95% CI	Z-score	p-value
KCNJ11*aa*5	**E**V**LI**S	0.647	0.616			1			
KCNJ11*aa*8	**K**V**LV**S	0.336	0.365	0.13	0.04	1.14	(1.05,1.24)	2.99	0.003
KCNJ11*aa*9	**K**V**VV**S	0.015	0.018	0.21	0.16	1.24	(0.9, 1.69)	1.32	0.188
**Uncommon**									
KCNJ11*aa*4	**E**V**LI**T	0.001	0.0003						
KCNJ11*aa*1	**E**L**LIS**	0.0005	0						

By converting the predicted genomic gene allele to amino acid sequences, the total number of gene alleles is reduced to 5, with four amino acid variations (**E23K**, L64V, **L270V**, **I337V**, and S385C). The 3 common haplotypes were considered in this case-control study.

*The amino acid variations among the three common haplotypes are highlighted in bold.

## Discussion

The 1000 Genomes Project, built upon the success of the HapMap project, promises to generate high quality sequence data for thousands of subjects from multiple racial/ethnic groups. Besides shedding light on human genome variations, this project will also generate an abundance of sequence data that will be useful for fine mapping and better defining disease associated functional variants. Towards this goal, we have described a method for in silico sequencing of candidate genes and regions of potential interest. Through training and validation exercises on currently available sequence data from the 1000 Genomes project, we have shown that the method produces reliable prediction models with high accuracies, with few exceptions. To facilitate the future use for the research community, we developed a set of predictive models for 611 genes utilizing all available sequence data. Such models can now be used to sequence known candidate genes in silico and further to investigate their functional properties, prior to carrying out actual sequencing in the wet lab. Therefore, applications of this method would help accelerate validation researches from discoveries to functional characterization in the most cost-effective and time-efficient way.

### Connection with SNP Imputation Methods

Our method of predicting polymorphic genetic variants may be thought of as an extension to imputation methods. Specifically, like imputation methods, our method also uses local LD to infer untyped gene alleles (or equivalently, phased diplotypes with multiple SNPs), whereas imputation methods infer untyped SNPs. By predicting fully phased sequence diploids, our approach allows one to translate DNA sequence variants in the coding region into amino acid sequence variants, thereby facilitating the functional interpretation of disease associations.

### Connection with Haplotype based Association Analysis

Our methodology also helps to resolve a long-standing debate on the value of haplotype based association analysis. Recall that haplotype based association analysis, since its inception, has been suggested as a preferred method for assessing disease association [19-22], because of its desired genetic interpretation and reduced number of multiple comparisons. However, the broader application of haplotype based association analyses would require investigators to rationally choose "haplotype units" of multiple SNPs, such as "haplotype blocks" [19,23,24]. Further investigation of the human genome has suggested that such blocks are not as robust as they were originally postulated and are also variable across populations. Thus, "haplotype unit" remains to be conceptually appealing. In practice, the majority of GWAS takes simple SNP-by-SNP association analysis, based upon publications thus far, because SNP-based association analysis is straightforward and is more amenable to comparisons across studies. Our proposed analytic strategy potentially has the advantages from both sides: 1) it focuses on natural units such as coding genes, or functional regions, which are comparable across studies; 2) it facilitates interpretations by framing associations in terms of diploid sequences and reduces the severity of multiple comparison issue.

### Structural Variants

The current methodology can naturally incorporate structural variants in building prediction models. One of the major advantages of direct sequencing is the ability to identify structural variants, including insertions and deletions. Structural variants could be naturally coded as additional gene alleles, and incorporated into prediction models. In the current study, few genes are known to have structural variants in those sequenced exons; no illustrations are given here.

### Choice of the Flanking Regions

During the model training process, one subjective parameter to be chosen, is the flanking region size for each gene (or region). The larger the flanking region, the more likely it is that all predictive SNPs are included, but also the greater is the chance that the prediction models will be over-fitting. Based upon our empirical experiences determining the optimal size of flanking regions for predicting alleles for five HLA genes [3,15], we have concluded that a default 150 KB flanking region is usually satisfactory. However, since the majority of the genes modeled for this study are larger than HLA genes, the empirical data summarized in Figure 3 lead us to use a flanking region of ± 250 kb for each gene.

### Limitation 1: poor prediction accuracies

While prediction accuracies of most genes are approaching 99%, it is noted that two genes (TLL1 and ZNF474) have particularly low prediction accuracies in the validation set. At CT = 0, the lowest prediction accuracy among all the 611 genes is 79% for TLL1 gene. This gene is one of the complex genes in this dataset (see Figure 2), with length of 230,584, as many as 31 SNPs, more than 50 alleles and entropy of 1.38. More than half of the prediction errors made on the validation set occur for uncommon alleles with frequency < 0.01. The predictive model includes 28 SNPs, with the prediction accuracy of 97% and 79% on the training and validation sets respectively, which indicates a possible model over-fitting. The remedies for such a problem include 1) increasing sample size in the training set and thus improving the power of predicting gene alleles, or 2) reducing the gene complexity by dividing the single gene into two or more segments based on their LD structure, or 3) choosing a smaller size of searching boundary when training the model.

The second gene ZNF474 has the prediction accuracy of 80% on the training set. Seventy out of the 92 prediction errors involved three alleles ZNF474*1, ZNF474*2 and ZNF474*3, which differ by two SNPs (rs2560306 and rs35262183). Unfortunately, neither SNP was genotyped nor in significant LD with any of the SNPs in the HapMap 3. In the absence of these "highly informative and yet isolated SNPs", one would not be able to differentiate these alleles. This phenomenon has also been observed for HLA predictions [3]. To overcome this limitation, the remedy is to restrict the prediction only to the combined alleles, a common practice in HLA genotyping. If separating such alleles is essential for practical reasons in prospective studies, one has no choice but to genotype additional missing SNPs.

### Limitation 2: poor diploid sequence quality

In order to recover diploid sequences from the 1000 Genomes Project, it is necessary to statistically infer for haplotypes. Among the 645 selected genes, 32 genes have been excluded, because > 10% of samples had relatively poor haplotype inference with probability less than 0.95. The poorer haplotype inference may associate with a complex gene structure. For example, of these 32 genes, four genes (ANKRD15, TRPV3, NLRP11, CYP24A1) are longer than 850Kb, or the entropies are greater than 2, or have more than 50 gene alleles. To address this challenge, our strategy is to divide a gene into several regions, e.g., by combining adjacent exons or by short segments, and then deduce the alleles within each region separately. On the other hand, for those genes with complex structural variations, probably the most effective approach is to use sequence technologies that produce relatively long reads, such as several hundred or even thousand nucleotide bases, which allow us to deduce diploid sequences directly. Technological improvement in the future would lessen the impact of this particular limitation.

### Limitation 3: limited polymorphisms in the training samples

A critical factor of building useful prediction models, especially for those genes with rare gene alleles, is to have a sufficiently large sample size so that rare alleles are observed in the training set. In our study, the SNP and sequence data used in this paper came from the 614 samples in both HapMap and the 1000 Genomes Project. Prediction accuracies should be improved when much more samples are available in future.

## Conclusions

Prior to the general availability of routine sequencing all subjects, the ISS method proposed here provides a timely and cost-effective approach to broadening the characterization of disease associated SNPs and regions, and facilitating the prioritization of candidate genes for more detailed functional and mechanistic studies.

## Authors' contributions

X.C.Z. participated in the initial discussion of this project, performed all data analysis, and participated in drafting the manuscript; B.Z. contributed to the development of ISS software. S.S.L. contributed to assisting the data analysis, and to preparation of the manuscript; X.H. contributed to the discussion of methodology; J.A.H. contributed to the original idea, and helped the manuscript drafting; L.P.Z. conceived the idea, and lead the project. All authors read and approved the final manuscript.

## Supplementary Material

Additional file 1 **Supplement Methods**. SNP selection process in building prediction models. Click here for file

Additional file 2 **Supplement Table 1**. Genes and their Properties. Click here for file

Additional file 3 **Supplement Table 2**. Accuracy % (call rate %) of the prediction models evaluated on training and validation set. Click here for file
