# Supplementary material for: Sequencing genes in silico using single nucleotide polymorphisms
Source: BMC Genet. 2012 Jan 30;13:6. doi: 10.1186/1471-2156-13-6 (PMC3283449; doi:10.1186/1471-2156-13-6)
Supplement: Additional file 1 — Supplement Methods. SNP selection process in building prediction models. [file 1471-2156-13-6-S1.DOCX]

# Supplemental Methods

In this study, we have expanded the multi-allelic genes (MAG) prediction methodology to predict alleles of genes sequenced in the 1000 Genomes Project Pilot3. Since some of the genes under consideration extend as long as 0.9Mb or with as many as 42 SNPs, we have incorporated several modifications as follows to ensure computational efficiency and feasibility.

Step 1. Include all SNPs inside and at most 1 flanking SNP on either side of each target region in the gene in the initial set of SNPs (S). Calculate the objective function and denote as Q^0^. Denote the non-empty set of SNPs on the left of each target region as LS = (LS_1_, LS_2_,…,LS_l_), and the one on the right as RS = (RS_1_, RS_2_,…,RS_r_).

Step 2. Perform a backward elimination as in Step 3b. Repeat this step until no further SNP needs to be removed.

Step 3. Take the right most SNP from each set LS_i_, perform Step3f and 3b, and update RS.

Step 3f. Perform a forward selection by adding one more SNP to S and calculate the objective function, which is denoted as Q^1^. If Q^1^<Q^0^, add this SNP to S, set Q^0^= Q^1^, and perform Step 3b.

Step 3b. Perform a backward elimination by removing each SNP s in S and calculate the objective function Q(s). If min(Q(s), s ∈S)≤Q^0^, remove the SNP s with the minimum Q(s) from S and reset Q^0^=min(Q(s), s ∈S).

Step 4. Take the left most SNP from each set RS_i_, perform Step3f and 3b, and update LS.

Step 5. Repeat steps 3-4 until the pre-set boundary on both sides is reached.
