# Supplementary material for: Sequencing genes in silico using single nucleotide polymorphisms
Source: BMC Genet. 2012 Jan 30;13:6. doi: 10.1186/1471-2156-13-6 (PMC3283449; doi:10.1186/1471-2156-13-6)
Supplement: Additional file 2 — Supplement Table 1. Genes and their Properties. [file 1471-2156-13-6-S2.DOCX]

| **Supplement Table 1: Genes and their Properties** | | | |  |  |  |
| --- | --- | --- | --- | --- | --- | --- |
| **Gene Name** | **Chr** | **Position** | **Length** | **N SNPs** | **N Alleles** | **Entropy** |
| AASDHPPT | 11 | 105453501-105474629 | 21128 | 3 | 4 | 0.1 |
| AATF | 17 | 32380287-32488284 | 107997 | 21 | 20 | 0.86 |
| ABCB9 | 12 | 121979491-122025705 | 46214 | 18 | 19 | 0.28 |
| ABLIM3 | 5 | 148501246-148620192 | 118946 | 15 | 17 | 0.42 |
| ABTB2 | 11 | 34129110-34335378 | 206268 | 14 | 22 | 0.84 |
| ACN9 | 7 | 96583840-96649011 | 65171 | 4 | 5 | 0.21 |
| ACOX3 | 4 | 8418908-8493352 | 74444 | 25 | 21 | 0.91 |
| ACPL2 | 3 | 142433371-142496176 | 62805 | 14 | 17 | 0.89 |
| ACTL7B | 9 | 110656691-110658031 | 1340 | 18 | 19 | 1.37 |
| ADAM29 | 4 | 176076133-176135906 | 59773 | 18 | 14 | 0.7 |
| ADD2 | 2 | 70688257-70848837 | 160580 | 11 | 12 | 0.25 |
| AGPAT3 | 21 | 44109543-44231903 | 122360 | 12 | 14 | 0.27 |
| AGPAT4 | 6 | 161471046-161615097 | 144051 | 11 | 11 | 0.64 |
| AHSA2 | 2 | 61258056-61267562 | 9506 | 4 | 5 | 0.11 |
| AIF1 | 6 | 31691011-31692777 | 1766 | 2 | 3 | 0.4 |
| AIG1 | 6 | 143422192-143703134 | 280942 | 7 | 6 | 0.66 |
| AK7 | 14 | 95928200-96024865 | 96665 | 26 | 29 | 1.12 |
| AKAP7 | 6 | 131508153-131646366 | 138213 | 10 | 14 | 0.67 |
| AKR1B1 | 7 | 133777646-133794428 | 16782 | 9 | 10 | 0.2 |
| AKR1CL1 | 10 | 5193674-5217144 | 23470 | 7 | 9 | 0.62 |
| AKR1D1 | 7 | 137411735-137453590 | 41855 | 5 | 6 | 0.11 |
| ALDH1A2 | 15 | 56032918-56145198 | 112280 | 13 | 12 | 0.83 |
| ALS2CR13 | 2 | 203208455-203342725 | 134270 | 5 | 7 | 0.09 |
| AMPD2 | 1 | 109963648-109976200 | 12552 | 10 | 10 | 0.5 |
| ANAPC5 | 12 | 120230542-120276395 | 45853 | 20 | 21 | 0.35 |
| ANKRD12 | 18 | 9126757-9275206 | 148449 | 20 | 16 | 0.9 |
| ANP32C | 4 | 165337608-165338313 | 705 | 20 | 23 | 1.14 |
| APCDD1 | 18 | 10444624-10478698 | 34074 | 19 | 19 | 1.21 |
| APITD1 | 1 | 10412745-10434797 | 22052 | 2 | 3 | 0.02 |
| APOBEC3F | 22 | 37747063-37781921 | 34858 | 2 | 3 | 0.77 |
| AQP3 | 9 | 33431153-33437590 | 6437 | 6 | 7 | 0.66 |
| ARL6IP6 | 2 | 153283375-153325669 | 42294 | 3 | 4 | 0.03 |
| ARNT | 1 | 149048809-149115810 | 67001 | 10 | 11 | 0.78 |
| ASAH2 | 10 | 51617077-51678319 | 61242 | 6 | 8 | 0.93 |
| ASL | 7 | 65178210-65195764 | 17554 | 6 | 7 | 0.09 |
| ATG5 | 6 | 106739044-106880388 | 141344 | 3 | 4 | 0.13 |
| ATHL1 | 11 | 279137-285688 | 6551 | 15 | 17 | 1.55 |
| ATP2B1 | 12 | 88505956-88573975 | 68019 | 11 | 13 | 0.61 |
| ATP2C1 | 3 | 132095711-132218245 | 122534 | 12 | 13 | 0.61 |
| ATRNL1 | 10 | 116843113-117698486 | 855373 | 13 | 17 | 1.19 |
| B3GALTL | 13 | 30672111-30804411 | 132300 | 11 | 12 | 0.82 |
| B4GALT6 | 18 | 27456206-27518684 | 62478 | 9 | 8 | 0.15 |
| BAG2 | 6 | 57145292-57157694 | 12402 | 2 | 3 | 0.01 |
| BAZ1A | 14 | 34291687-34414604 | 122917 | 19 | 18 | 1.13 |
| BCAP29 | 7 | 107007945-107058401 | 50456 | 3 | 4 | 0.02 |
| BCHE | 3 | 166973385-167037947 | 64562 | 23 | 19 | 0.6 |
| BCL11A | 2 | 60531805-60634137 | 102332 | 13 | 11 | 0.67 |
| BCL2L11 | 2 | 111594961-111642493 | 47532 | 3 | 4 | 0.51 |
| BDH1 | 3 | 198721050-198784591 | 63541 | 12 | 17 | 0.47 |
| BLMH | 17 | 25599348-25643200 | 43852 | 5 | 8 | 0.67 |
| BOLL | 2 | 198299847-198359183 | 59336 | 3 | 4 | 0.11 |
| BRD7 | 16 | 48910441-48960330 | 49889 | 13 | 13 | 0.27 |
| BRMS1L | 14 | 35365347-35410920 | 45573 | 3 | 4 | 0.04 |
| BTBD1 | 15 | 81476184-81527110 | 50926 | 7 | 7 | 0.1 |
| BXDC2 | 5 | 34951576-34961545 | 9969 | 10 | 9 | 0.19 |
| C10orf65 | 10 | 99334110-99362545 | 28435 | 11 | 12 | 0.88 |
| C10orf78 | 10 | 105871805-105876133 | 4328 | 3 | 4 | 0.57 |
| C11orf53 | 11 | 110631916-110662182 | 30266 | 8 | 9 | 0.32 |
| C12orf63 | 12 | 95565883-95683179 | 117296 | 30 | 27 | 1.81 |
| C14orf1 | 14 | 75185883-75196912 | 11029 | 4 | 5 | 0.04 |
| C14orf108 | 14 | 56805379-56826545 | 21166 | 14 | 7 | 0.28 |
| C15orf40 | 15 | 81464407-81471362 | 6955 | 5 | 6 | 0.07 |
| C16orf48 | 16 | 66254350-66258129 | 3779 | 8 | 9 | 0.13 |
| C16orf55 | 16 | 88251710-88265176 | 13466 | 8 | 8 | 0.21 |
| C17orf25 | 17 | 609321-632297 | 22976 | 20 | 16 | 0.37 |
| C17orf53 | 17 | 39574851-39595370 | 20519 | 13 | 12 | 0.83 |
| C17orf66 | 17 | 31206072-31220008 | 13936 | 19 | 21 | 0.98 |
| C17orf79 | 17 | 27203011-27210369 | 7358 | 4 | 5 | 0.36 |
| C18orf10 | 18 | 32620469-32663156 | 42687 | 6 | 7 | 0.66 |
| C19orf26 | 19 | 1180946-1188990 | 8044 | 2 | 3 | 0.27 |
| C1QBP | 17 | 5276822-5283195 | 6373 | 4 | 5 | 0.05 |
| C1orf178 | 1 | 114220962-114231726 | 10764 | 8 | 9 | 0.08 |
| C1orf198 | 1 | 229039488-229071958 | 32470 | 10 | 7 | 0.37 |
| C1orf63 | 1 | 25441326-25446572 | 5246 | 8 | 9 | 0.52 |
| C1orf9 | 1 | 170768882-170847596 | 78714 | 27 | 20 | 0.88 |
| C1orf94 | 1 | 34405210-34457318 | 52108 | 17 | 23 | 1.31 |
| C2orf18 | 2 | 26840645-26857603 | 16958 | 4 | 5 | 0.12 |
| C2orf47 | 2 | 200528284-200537085 | 8801 | 4 | 5 | 0.06 |
| C3orf14 | 3 | 62279750-62294360 | 14610 | 3 | 4 | 0.08 |
| C3orf37 | 3 | 130480373-130506825 | 26452 | 6 | 7 | 0.39 |
| C3orf38 | 3 | 88281798-88288755 | 6957 | 5 | 6 | 0.6 |
| C4orf33 | 4 | 130234278-130253293 | 19015 | 8 | 11 | 1.26 |
| C5orf25 | 5 | 175598008-175705596 | 107588 | 8 | 9 | 0.44 |
| C6orf117 | 6 | 84800139-84857320 | 57181 | 8 | 10 | 0.94 |
| C6orf130 | 6 | 41142508-41148246 | 5738 | 5 | 6 | 0.03 |
| C6orf145 | 6 | 3667834-3697245 | 29411 | 4 | 5 | 0.68 |
| C6orf51 | 6 | 111386456-111395772 | 9316 | 3 | 4 | 0.02 |
| C9orf131 | 9 | 35031101-35035988 | 4887 | 38 | 29 | 1.58 |
| CALML5 | 10 | 5530659-5531510 | 851 | 6 | 9 | 0.98 |
| CAPNS1 | 19 | 41322757-41333095 | 10338 | 4 | 5 | 0.09 |
| CART1 | 12 | 84198015-84219690 | 21675 | 7 | 9 | 0.24 |
| CASP10 | 2 | 201755865-201802355 | 46490 | 10 | 11 | 1.03 |
| CASP7 | 10 | 115428924-115480654 | 51730 | 13 | 15 | 1.02 |
| CBLB | 3 | 106859798-107070577 | 210779 | 35 | 43 | 1.48 |
| CCBE1 | 18 | 55252126-55515570 | 263444 | 11 | 13 | 0.13 |
| CCDC63 | 12 | 109769193-109829721 | 60528 | 24 | 25 | 0.64 |
| CCK | 3 | 42274321-42282666 | 8345 | 2 | 3 | 0.03 |
| CCKAR | 4 | 26092115-26101140 | 9025 | 17 | 16 | 0.35 |
| CCNDBP1 | 15 | 41264831-41274687 | 9856 | 7 | 8 | 0.14 |
| CCT6B | 17 | 30279053-30312619 | 33566 | 17 | 23 | 1.32 |
| CD1C | 1 | 156526186-156531188 | 5002 | 14 | 13 | 0.63 |
| CD300LB | 17 | 70028907-70039208 | 10301 | 5 | 6 | 0.64 |
| CD46 | 1 | 205992024-206035481 | 43457 | 6 | 7 | 0.09 |
| CD79B | 17 | 59359829-59363436 | 3607 | 7 | 8 | 0.77 |
| CD9 | 12 | 6179133-6217688 | 38555 | 6 | 8 | 0.11 |
| CDC25C | 5 | 137648857-137701943 | 53086 | 10 | 10 | 0.11 |
| CDCA2 | 8 | 25372429-25421342 | 48913 | 40 | 26 | 1.59 |
| CDKN2A | 9 | 21957750-21984490 | 26740 | 3 | 4 | 0.07 |
| CENPH | 5 | 68521130-68541940 | 20810 | 2 | 3 | 0.03 |
| CEP250 | 20 | 33506563-33563217 | 56654 | 42 | 47 | 1.47 |
| CETN1 | 18 | 570368-571524 | 1156 | 8 | 9 | 0.67 |
| CHEK1 | 11 | 125000245-125051360 | 51115 | 3 | 4 | 0.07 |
| CHFR | 12 | 131927010-131974257 | 47247 | 16 | 14 | 1.57 |
| CIDEA | 18 | 12244317-12267594 | 23277 | 7 | 8 | 0.8 |
| CLDN10 | 13 | 94883858-95029907 | 146049 | 6 | 6 | 0.09 |
| CLDN19 | 1 | 42971350-42978512 | 7162 | 3 | 4 | 0.17 |
| CLEC2D | 12 | 9713575-9743306 | 29731 | 4 | 7 | 1.32 |
| CLRN3 | 10 | 129566103-129581201 | 15098 | 9 | 10 | 0.32 |
| CLSPN | 1 | 35974404-36008138 | 33734 | 36 | 26 | 1.28 |
| CMPK | 1 | 47572174-47617097 | 44923 | 2 | 3 | 0.34 |
| CNTFR | 9 | 34541430-34579722 | 38292 | 5 | 6 | 0.17 |
| COG7 | 16 | 23307316-23372004 | 64688 | 25 | 19 | 0.5 |
| COL4A3BP | 5 | 74705735-74843719 | 137984 | 7 | 8 | 0.66 |
| COLQ | 3 | 15466643-15538262 | 71619 | 11 | 12 | 0.54 |
| COMMD9 | 11 | 36250417-36267575 | 17158 | 2 | 3 | 0.03 |
| COPS8 | 2 | 237658822-237672228 | 13406 | 2 | 3 | 0.02 |
| CRB1 | 1 | 195504030-195714208 | 210178 | 24 | 26 | 0.66 |
| CREB3L2 | 7 | 137210264-137337386 | 127122 | 8 | 8 | 0.79 |
| CREB5 | 7 | 28305464-28832036 | 526572 | 7 | 8 | 0.08 |
| CRELD1 | 3 | 9950505-9962090 | 11585 | 10 | 9 | 0.37 |
| CRNN | 1 | 150648342-150653352 | 5010 | 15 | 16 | 1.07 |
| CRYGA | 2 | 208733708-208736542 | 2834 | 7 | 8 | 0.08 |
| CTDSPL | 3 | 37878672-38000964 | 122292 | 6 | 7 | 0.1 |
| CTSS | 1 | 148969175-149004929 | 35754 | 3 | 4 | 0.68 |
| CXCL13 | 4 | 78651929-78752010 | 100081 | 2 | 3 | 0.03 |
| CYB561D1 | 1 | 109838244-109844585 | 6341 | 6 | 6 | 0.13 |
| CYBASC3 | 11 | 60872853-60886305 | 13452 | 5 | 6 | 0.07 |
| CYP2A13 | 19 | 46286207-46293939 | 7732 | 2 | 3 | 0.05 |
| CYP2C18 | 10 | 96433240-96485937 | 52697 | 16 | 16 | 1 |
| CYP3A7 | 7 | 99140595-99170757 | 30162 | 10 | 9 | 0.89 |
| CYP4X1 | 1 | 47199622-47289010 | 89388 | 10 | 12 | 0.13 |
| CYP7B1 | 8 | 65671082-65873902 | 202820 | 8 | 9 | 0.13 |
| DAZAP2 | 12 | 49918892-49923834 | 4942 | 3 | 4 | 0.03 |
| DCLRE1C | 10 | 14988876-15036100 | 47224 | 18 | 19 | 1.45 |
| DCTN5 | 16 | 23560307-23588683 | 28376 | 4 | 5 | 0.86 |
| DDI1 | 11 | 103412517-103415132 | 2615 | 18 | 18 | 1.08 |
| DDX49 | 19 | 18891493-18900436 | 8943 | 16 | 16 | 0.52 |
| DDX50 | 10 | 70331039-70376609 | 45570 | 8 | 8 | 0.26 |
| DGKZ | 11 | 46311314-46358680 | 47366 | 6 | 7 | 0.15 |
| DHRS7B | 17 | 20970849-21035428 | 64579 | 9 | 9 | 0.22 |
| DKK4 | 8 | 42350742-42353831 | 3089 | 6 | 8 | 0.69 |
| DLGAP4 | 20 | 34357716-34590454 | 232738 | 11 | 12 | 0.42 |
| DNAJC15 | 13 | 42495361-42581306 | 85945 | 5 | 6 | 0.59 |
| DUSP10 | 1 | 219941388-219982084 | 40696 | 4 | 5 | 0.06 |
| DUSP26 | 8 | 33568392-33576981 | 8589 | 8 | 8 | 0.35 |
| DYX1C1 | 15 | 53497245-53587724 | 90479 | 5 | 6 | 0.07 |
| EFCAB3 | 17 | 57811645-57847569 | 35924 | 10 | 8 | 0.2 |
| ELAVL4 | 1 | 50286375-50439643 | 153268 | 7 | 5 | 0.85 |
| EMCN | 4 | 101538006-101658202 | 120196 | 6 | 7 | 0.19 |
| ENDOG | 9 | 130620599-130624776 | 4177 | 6 | 5 | 0.58 |
| ENTHD1 | 22 | 38468994-38619740 | 150746 | 20 | 19 | 0.36 |
| EPB42 | 15 | 41276717-41300615 | 23898 | 19 | 22 | 0.49 |
| EPN2 | 17 | 19081282-19180621 | 99339 | 10 | 11 | 0.16 |
| EPYC | 12 | 89881589-89922934 | 41345 | 13 | 13 | 0.27 |
| ERCC3 | 2 | 127731335-127768222 | 36887 | 17 | 17 | 0.36 |
| ESAM | 11 | 124128228-124137433 | 9205 | 10 | 11 | 0.36 |
| ETS2 | 21 | 39099100-39118748 | 19648 | 18 | 16 | 1 |
| ETV5 | 3 | 187246803-187309571 | 62768 | 10 | 10 | 0.21 |
| EXTL1 | 1 | 26220857-26235541 | 14684 | 15 | 14 | 1.51 |
| F2RL1 | 5 | 76150609-76166896 | 16287 | 10 | 12 | 0.75 |
| FADS3 | 11 | 61397573-61415998 | 18425 | 6 | 5 | 0.05 |
| FAM105A | 5 | 14634931-14664205 | 29274 | 13 | 14 | 0.24 |
| FAM112B | 12 | 53136011-53153614 | 17603 | 3 | 5 | 0.07 |
| FAM117A | 17 | 45142685-45196518 | 53833 | 13 | 15 | 0.44 |
| FAM19A3 | 1 | 113064711-113071379 | 6668 | 6 | 6 | 0.22 |
| FAM19A4 | 3 | 68863606-69064401 | 200795 | 3 | 4 | 0.05 |
| FAM73A | 1 | 78017896-78116669 | 98773 | 7 | 8 | 0.1 |
| FAS | 10 | 90740267-90765522 | 25255 | 11 | 14 | 0.8 |
| FASTKD5 | 20 | 3075164-3088540 | 13376 | 22 | 20 | 0.83 |
| FBXL4 | 6 | 99428321-99502570 | 74249 | 10 | 7 | 0.46 |
| FBXO24 | 7 | 100021891-100036676 | 14785 | 8 | 8 | 0.08 |
| FCF1 | 14 | 74249602-74273250 | 23648 | 4 | 5 | 0.11 |
| FGF3 | 11 | 69333916-69343129 | 9213 | 5 | 5 | 0.17 |
| FIBP | 11 | 65407786-65412586 | 4800 | 8 | 9 | 0.15 |
| FLJ10781 | 19 | 51661588-51666617 | 5029 | 18 | 21 | 1 |
| FLJ16793 | 5 | 132111035-132117755 | 6720 | 5 | 6 | 0.06 |
| FLJ31951 | 5 | 158516996-158567412 | 50416 | 4 | 5 | 0.02 |
| FLJ46688 | 12 | 91654441-91689999 | 35558 | 13 | 14 | 1.12 |
| FOXI1 | 5 | 169465494-169469307 | 3813 | 8 | 9 | 0.93 |
| FSHR | 2 | 49043155-49235134 | 191979 | 9 | 13 | 1.09 |
| FTSJ3 | 17 | 59250524-59258188 | 7664 | 31 | 31 | 1.14 |
| FXC1 | 11 | 6459252-6462487 | 3235 | 6 | 8 | 0.71 |
| FZD9 | 7 | 72486044-72488386 | 2342 | 8 | 10 | 0.14 |
| GAB2 | 11 | 77603989-77806414 | 202425 | 26 | 32 | 1.28 |
| GABPA | 21 | 26028751-26066642 | 37891 | 4 | 6 | 0.19 |
| GALM | 2 | 38746742-38815031 | 68289 | 10 | 11 | 0.71 |
| GALNT13 | 2 | 154436672-155018735 | 582063 | 7 | 7 | 0.14 |
| GATAD2A | 19 | 19357641-19480741 | 123100 | 8 | 8 | 0.76 |
| GBP6 | 1 | 89602023-89626307 | 24284 | 30 | 27 | 1.43 |
| GDNF | 5 | 37851509-37875539 | 24030 | 5 | 5 | 0.12 |
| GEFT | 12 | 56290229-56297293 | 7064 | 14 | 14 | 1.21 |
| GENX-3414 | 4 | 77446832-77451306 | 4474 | 8 | 8 | 0.18 |
| GGH | 8 | 64090192-64113940 | 23748 | 5 | 6 | 0.31 |
| GIMAP7 | 7 | 149842877-149849094 | 6217 | 14 | 17 | 1.59 |
| GJA5 | 1 | 145694955-145712108 | 17153 | 3 | 4 | 0.14 |
| GLOXD1 | 1 | 45565131-45566931 | 1800 | 9 | 9 | 0.18 |
| GLS2 | 12 | 55151002-55168448 | 17446 | 9 | 11 | 0.59 |
| GMCL1 | 2 | 69910321-69960231 | 49910 | 5 | 6 | 0.15 |
| GNG4 | 1 | 233777607-233880677 | 103070 | 2 | 3 | 0.69 |
| GOPC | 6 | 117746043-118030374 | 284331 | 8 | 9 | 0.06 |
| GPD2 | 2 | 157000210-157178493 | 178283 | 18 | 16 | 1.02 |
| GPR114 | 16 | 56134057-56168601 | 34544 | 21 | 13 | 1.3 |
| GPR120 | 10 | 95316411-95337356 | 20945 | 7 | 7 | 0.15 |
| GPR128 | 3 | 101811134-101896955 | 85821 | 27 | 25 | 1.1 |
| GPR37L1 | 1 | 200358651-200365257 | 6606 | 13 | 16 | 1.25 |
| GPT2 | 16 | 45475808-45522699 | 46891 | 15 | 13 | 0.4 |
| GPX7 | 1 | 52840631-52847310 | 6679 | 4 | 5 | 0.56 |
| GRIA4 | 11 | 104986009-105358029 | 372020 | 11 | 11 | 0.33 |
| GTDC1 | 2 | 144420050-144806499 | 386449 | 16 | 16 | 0.85 |
| GTF2E2 | 8 | 30555572-30635280 | 79708 | 5 | 6 | 0.05 |
| GTF2H5 | 6 | 158509371-158533364 | 23993 | 2 | 3 | 0.01 |
| GUF1 | 4 | 44375189-44397454 | 22265 | 10 | 11 | 0.79 |
| H2AFY2 | 10 | 71482362-71542046 | 59684 | 14 | 14 | 0.17 |
| HAGH | 16 | 1799104-1817196 | 18092 | 11 | 12 | 0.53 |
| HAX1 | 1 | 152511662-152514975 | 3313 | 2 | 3 | 0.01 |
| HFE | 6 | 26195487-26205035 | 9548 | 3 | 4 | 0.05 |
| HIF3A | 19 | 51492144-51538530 | 46386 | 12 | 13 | 1.2 |
| HIGD2A | 5 | 175748389-175749357 | 968 | 2 | 3 | 0.27 |
| HINT3 | 6 | 126319553-126343082 | 23529 | 2 | 2 | 0.55 |
| HIPK1 | 1 | 114273518-114321945 | 48427 | 13 | 15 | 1.16 |
| HIRA | 22 | 17698223-17815220 | 116997 | 13 | 12 | 0.22 |
| HISPPD1 | 5 | 102483856-102566808 | 82952 | 26 | 28 | 1.05 |
| HIST1H2BC | 6 | 26222609-26232133 | 9524 | 3 | 4 | 0.02 |
| HIST1H2BD | 6 | 26266327-26279555 | 13228 | 7 | 8 | 0.19 |
| HIST1H2BJ | 6 | 27200740-27208554 | 7814 | 11 | 12 | 0.13 |
| HIST1H2BO | 6 | 27969181-27969648 | 467 | 2 | 3 | 0.01 |
| HMGN4 | 6 | 26646550-26655143 | 8593 | 4 | 5 | 0.75 |
| HNRPH1 | 5 | 178973785-178983276 | 9491 | 10 | 11 | 0.23 |
| HNRPK | 9 | 85772818-85785339 | 12521 | 5 | 6 | 0.05 |
| HRH4 | 18 | 20294590-20313919 | 19329 | 10 | 11 | 0.51 |
| HSCB | 22 | 27468019-27483503 | 15484 | 3 | 4 | 0.17 |
| HSD17B3 | 9 | 98037409-98104255 | 66846 | 12 | 12 | 0.65 |
| HSD3B2 | 1 | 119758792-119790643 | 31851 | 13 | 14 | 0.31 |
| HSP90AA1 | 14 | 101616827-101675839 | 59012 | 23 | 23 | 1.44 |
| HSPA14 | 10 | 14920266-14953746 | 33480 | 9 | 8 | 0.07 |
| HTATIP | 11 | 65236065-65243650 | 7585 | 6 | 7 | 0.13 |
| HTR7 | 10 | 92490555-92607651 | 117096 | 5 | 6 | 0.18 |
| ICAM4 | 19 | 10258649-10260198 | 1549 | 2 | 3 | 0.04 |
| IFI44 | 1 | 78888064-78902351 | 14287 | 17 | 16 | 0.53 |
| IFNGR2 | 21 | 33697071-33731698 | 34627 | 8 | 9 | 0.89 |
| IKZF3 | 17 | 35174724-35273967 | 99243 | 10 | 9 | 0.73 |
| IL15 | 4 | 142777203-142874062 | 96859 | 4 | 5 | 0.05 |
| IL24 | 1 | 205137411-205144107 | 6696 | 4 | 5 | 0.78 |
| IMP3 | 15 | 73718483-73728102 | 9619 | 2 | 3 | 0.68 |
| IMPACT | 18 | 20260606-20287492 | 26886 | 4 | 5 | 0.7 |
| INSIG1 | 7 | 154720475-154732879 | 12404 | 4 | 5 | 0.18 |
| INVS | 9 | 101901331-102103247 | 201916 | 29 | 16 | 0.86 |
| IRAK4 | 12 | 42439019-42468166 | 29147 | 7 | 8 | 0.07 |
| IRF4 | 6 | 336751-356443 | 19692 | 14 | 13 | 0.43 |
| IRF6 | 1 | 208027884-208046105 | 18221 | 12 | 15 | 1.1 |
| ITGB4BP | 20 | 33330139-33336008 | 5869 | 2 | 3 | 0.05 |
| JOSD1 | 22 | 37411571-37426215 | 14644 | 5 | 6 | 0.52 |
| KCNJ11 | 11 | 17363371-17367440 | 4069 | 17 | 18 | 1.35 |
| KCNK12 | 2 | 47601418-47650974 | 49556 | 2 | 3 | 0.03 |
| KCNK13 | 14 | 89597860-89721948 | 124088 | 15 | 18 | 0.55 |
| KIAA0737 | 14 | 21015174-21037159 | 21985 | 6 | 8 | 0.75 |
| KIAA1446 | 14 | 100073240-100105884 | 32644 | 4 | 5 | 0.34 |
| KIAA1622 | 14 | 93710401-93815825 | 105424 | 11 | 10 | 0.31 |
| KIF9 | 3 | 47244888-47299092 | 54204 | 25 | 26 | 1.16 |
| KLHL12 | 1 | 201126852-201162994 | 36142 | 3 | 4 | 0.03 |
| KLHL31 | 6 | 53620657-53638465 | 17808 | 19 | 19 | 1.58 |
| KRR1 | 12 | 74177685-74191685 | 14000 | 9 | 12 | 0.83 |
| KRT18 | 12 | 51628921-51632952 | 4031 | 2 | 3 | 0.03 |
| KRT27 | 17 | 36186585-36192312 | 5727 | 19 | 31 | 2.02 |
| LASS3 | 15 | 98758122-98902448 | 144326 | 16 | 17 | 0.92 |
| LASS4 | 19 | 8180256-8233302 | 53046 | 11 | 16 | 1.52 |
| LCE1F | 1 | 151015471-151015828 | 357 | 5 | 6 | 0.22 |
| LCN10 | 9 | 138752439-138757235 | 4796 | 6 | 7 | 0.59 |
| LEO1 | 15 | 50017513-50051250 | 33737 | 9 | 10 | 0.34 |
| LEPREL1 | 3 | 191157315-191322748 | 165433 | 21 | 19 | 1.25 |
| LGTN | 1 | 204831597-204852527 | 20930 | 13 | 8 | 0.41 |
| LHX6 | 9 | 124004678-124030840 | 26162 | 5 | 5 | 0.14 |
| LIMK1 | 7 | 73136091-73174790 | 38699 | 14 | 15 | 0.3 |
| LIMK2 | 22 | 29938249-30006066 | 67817 | 18 | 18 | 0.71 |
| LIPL1 | 10 | 90336498-90356712 | 20214 | 8 | 9 | 0.22 |
| LOC133308 | 4 | 104166799-104217379 | 50580 | 18 | 17 | 0.54 |
| LOC144097 | 11 | 63337498-63351765 | 14267 | 6 | 5 | 0.73 |
| LOC153222 | 5 | 172415975-172496567 | 80592 | 9 | 10 | 0.56 |
| LOC284402 | 19 | 39776185-39777330 | 1145 | 3 | 4 | 0.1 |
| LOC389541 | 7 | 99584477-99589761 | 5284 | 5 | 6 | 0.76 |
| LONRF2 | 2 | 100256184-100305627 | 49443 | 21 | 21 | 1.6 |
| LPL | 8 | 19840861-19869050 | 28189 | 17 | 24 | 1.26 |
| LRG1 | 19 | 4488226-4491036 | 2810 | 19 | 19 | 1.26 |
| LRMP | 12 | 25096507-25152536 | 56029 | 13 | 14 | 0.75 |
| LRRC20 | 10 | 71728734-71812388 | 83654 | 5 | 6 | 0.06 |
| LRRC23 | 12 | 6852993-6893667 | 40674 | 9 | 11 | 1.2 |
| LRRC52 | 1 | 163779867-163799822 | 19955 | 9 | 10 | 0.97 |
| LTB4DH | 9 | 113365073-113401922 | 36849 | 8 | 10 | 0.74 |
| LYPD2 | 8 | 143828629-143830954 | 2325 | 3 | 4 | 0.03 |
| LYPD6 | 2 | 149895358-150038384 | 143026 | 6 | 7 | 0.1 |
| MAP3K12 | 12 | 52160546-52179538 | 18992 | 11 | 11 | 0.09 |
| MAPK9 | 5 | 179595389-179651573 | 56184 | 11 | 12 | 0.09 |
| MASP1 | 3 | 188418631-188492446 | 73815 | 22 | 29 | 1.32 |
| MBD1 | 18 | 46047253-46062142 | 14889 | 10 | 11 | 1.05 |
| MCART1 | 9 | 37867571-37894350 | 26779 | 2 | 3 | 0.5 |
| MCMDC1 | 6 | 119273462-119298002 | 24540 | 13 | 15 | 0.81 |
| MELK | 9 | 36562904-36667679 | 104775 | 13 | 16 | 0.55 |
| MFAP1 | 15 | 41884024-41904243 | 20219 | 9 | 11 | 0.63 |
| MFSD2 | 1 | 40193396-40208215 | 14819 | 8 | 8 | 0.2 |
| MGC14376 | 17 | 1561553-1566254 | 4701 | 2 | 3 | 0.02 |
| MGC70857 | 8 | 145722410-145725266 | 2856 | 2 | 3 | 0.05 |
| MKL2 | 16 | 14072696-14268131 | 195435 | 22 | 21 | 0.73 |
| MOV10 | 1 | 113018714-113044882 | 26168 | 22 | 24 | 1.18 |
| MRCL3 | 18 | 3237527-3246234 | 8707 | 2 | 3 | 0.08 |
| MRPL39 | 21 | 25879838-25901672 | 21834 | 17 | 19 | 1.26 |
| MRPS18B | 6 | 30693464-30702153 | 8689 | 7 | 9 | 0.26 |
| MRPS7 | 17 | 70769343-70774052 | 4709 | 8 | 9 | 0.1 |
| MSH4 | 1 | 76035217-76151511 | 116294 | 15 | 12 | 1.56 |
| MTERFD2 | 2 | 241675181-241690397 | 15216 | 14 | 15 | 0.98 |
| MTMR2 | 11 | 95205693-95297019 | 91326 | 15 | 16 | 0.96 |
| MTP18 | 22 | 29135414-29155041 | 19627 | 4 | 6 | 0.73 |
| MYB | 6 | 135544145-135582003 | 37858 | 12 | 13 | 0.35 |
| MYEOV2 | 2 | 240714652-240724437 | 9785 | 9 | 9 | 1.23 |
| NAGLU | 17 | 37941476-37949992 | 8516 | 14 | 13 | 0.62 |
| NCKIPSD | 3 | 48686281-48698338 | 12057 | 8 | 8 | 0.16 |
| NCSTN | 1 | 158579686-158595366 | 15680 | 19 | 24 | 0.8 |
| NEK7 | 1 | 196392816-196555392 | 162576 | 7 | 6 | 0.09 |
| NEU1 | 6 | 31934807-31938688 | 3881 | 10 | 10 | 0.19 |
| NEUROD1 | 2 | 182249438-182253626 | 4188 | 8 | 9 | 0.57 |
| NFIA | 1 | 61320882-61694624 | 373742 | 11 | 13 | 0.78 |
| NFKBIZ | 3 | 103029546-103062556 | 33010 | 14 | 15 | 1.12 |
| NHN1 | 16 | 87164344-87225756 | 61412 | 21 | 22 | 0.7 |
| NKD1 | 16 | 49139741-49226142 | 86401 | 8 | 9 | 0.17 |
| NLF1 | 15 | 60146493-60148393 | 1900 | 2 | 3 | 0.1 |
| NMNAT3 | 3 | 140761722-140879530 | 117808 | 9 | 10 | 0.21 |
| NOL6 | 9 | 33451350-33463941 | 12591 | 40 | 38 | 1.12 |
| NOVA1 | 14 | 25984928-26136800 | 151872 | 2 | 3 | 0.02 |
| NPR1 | 1 | 151917787-151933092 | 15305 | 12 | 12 | 0.55 |
| NQO1 | 16 | 68300804-68318034 | 17230 | 6 | 7 | 0.86 |
| NRAS | 1 | 115048600-115061038 | 12438 | 2 | 3 | 0.01 |
| NSUN7 | 4 | 40446670-40506759 | 60089 | 10 | 11 | 0.71 |
| NT5C2 | 10 | 104837901-104943006 | 105105 | 7 | 9 | 0.9 |
| NUDT1 | 7 | 2248382-2257306 | 8924 | 8 | 8 | 0.52 |
| NUDT5 | 10 | 12249578-12278149 | 28571 | 5 | 5 | 0.75 |
| NUDT9 | 4 | 88562758-88598523 | 35765 | 9 | 9 | 0.43 |
| NUFIP1 | 13 | 44411383-44461613 | 50230 | 12 | 10 | 0.79 |
| NUMB | 14 | 72811670-72995039 | 183369 | 12 | 12 | 0.42 |
| NYD-SP18 | 7 | 128142678-128159033 | 16355 | 10 | 11 | 1.2 |
| OBP2B | 9 | 135070486-135074449 | 3963 | 2 | 3 | 0.04 |
| ODC1 | 2 | 10497958-10505904 | 7946 | 14 | 15 | 0.99 |
| OGG1 | 3 | 9765704-9783342 | 17638 | 5 | 5 | 0.03 |
| OLFM1 | 9 | 137106909-137152851 | 45942 | 9 | 10 | 0.69 |
| OPTC | 1 | 201729893-201744700 | 14807 | 14 | 15 | 0.28 |
| OR10J1 | 1 | 157676172-157677135 | 963 | 13 | 18 | 1.41 |
| OR13A1 | 10 | 45118107-45131062 | 12955 | 21 | 8 | 0.13 |
| OR2D3 | 11 | 6898808-6899801 | 993 | 18 | 19 | 1.77 |
| OR4D11 | 11 | 59027624-59028560 | 936 | 7 | 8 | 0.78 |
| OR51B4 | 11 | 5278819-5279752 | 933 | 22 | 25 | 1.67 |
| OR52H1 | 11 | 5522366-5523329 | 963 | 19 | 26 | 1.63 |
| OR56A4 | 11 | 5979856-5980954 | 1098 | 14 | 14 | 0.69 |
| OR6C76 | 12 | 54106304-54107243 | 939 | 15 | 20 | 0.85 |
| OR8A1 | 11 | 123945174-123946155 | 981 | 12 | 16 | 0.92 |
| OSGIN2 | 8 | 90983268-91009270 | 26002 | 8 | 9 | 0.28 |
| OXA1L | 14 | 22305570-22310607 | 5037 | 18 | 21 | 1.61 |
| P2RX2 | 12 | 131705475-131709045 | 3570 | 12 | 8 | 0.07 |
| PACS1 | 11 | 65594399-65768789 | 174390 | 13 | 14 | 0.32 |
| PAK1 | 11 | 76710707-76862756 | 152049 | 7 | 7 | 0.04 |
| PAK4 | 19 | 44308259-44361886 | 53627 | 5 | 6 | 0.11 |
| PAQR5 | 15 | 67378347-67487030 | 108683 | 10 | 12 | 0.67 |
| PAWR | 12 | 78509875-78608921 | 99046 | 3 | 4 | 0.6 |
| PAX4 | 7 | 127037581-127043218 | 5637 | 19 | 18 | 0.79 |
| PBK | 8 | 27723056-27751268 | 28212 | 9 | 12 | 1.31 |
| PBX3 | 9 | 127549486-127769476 | 219990 | 8 | 9 | 0.34 |
| PBX4 | 19 | 19533521-19590439 | 56918 | 3 | 4 | 0.04 |
| PCBD1 | 10 | 72313272-72318547 | 5275 | 2 | 3 | 0.02 |
| PCDH10 | 4 | 134289919-134332182 | 42263 | 11 | 13 | 0.5 |
| PCDHGC4 | 5 | 140844924-140872730 | 27806 | 11 | 11 | 0.29 |
| PCTK3 | 1 | 203740349-203768540 | 28191 | 12 | 13 | 0.89 |
| PDE8B | 5 | 76542461-76758984 | 216523 | 15 | 15 | 0.81 |
| PDGFB | 22 | 37949664-37970936 | 21272 | 6 | 9 | 0.18 |
| PDSS1 | 10 | 27026600-27075732 | 49132 | 6 | 7 | 0.11 |
| PDZD8 | 10 | 119032595-119124927 | 92332 | 25 | 20 | 0.91 |
| PF4 | 4 | 75065659-75066541 | 882 | 4 | 4 | 0.19 |
| PGBD1 | 6 | 28357342-28378305 | 20963 | 25 | 31 | 1.86 |
| PHF15 | 5 | 133889245-133946817 | 57572 | 19 | 19 | 0.55 |
| PHLDA3 | 1 | 199701244-199704922 | 3678 | 5 | 6 | 0.06 |
| PI4K2B | 4 | 24844772-24889808 | 45036 | 10 | 11 | 0.64 |
| PIAS2 | 18 | 42646057-42754121 | 108064 | 13 | 12 | 0.27 |
| PIGL | 17 | 16061233-16170298 | 109065 | 9 | 10 | 0.07 |
| PIGP | 21 | 37359533-37367328 | 7795 | 10 | 9 | 0.71 |
| PIGS | 17 | 23904531-23922655 | 18124 | 15 | 13 | 0.25 |
| PIK3R3 | 1 | 46278398-46371295 | 92897 | 8 | 8 | 0.84 |
| PIP5K2B | 17 | 34175470-34209684 | 34214 | 7 | 9 | 1.05 |
| PLAT | 8 | 42151392-42184351 | 32959 | 24 | 16 | 1.22 |
| PLCD1 | 3 | 38023990-38046137 | 22147 | 19 | 28 | 1.32 |
| PMM2 | 16 | 8799170-8861775 | 62605 | 5 | 6 | 0.13 |
| PMP22CD | 11 | 123258842-123261550 | 2708 | 11 | 11 | 0.61 |
| PODXL2 | 3 | 128830728-128874342 | 43614 | 15 | 16 | 0.68 |
| PON2 | 7 | 94872109-94902320 | 30211 | 12 | 14 | 0.69 |
| PPAT | 4 | 56954285-56996602 | 42317 | 5 | 5 | 0.67 |
| PPIG | 2 | 170149095-170202500 | 53405 | 16 | 14 | 1.31 |
| PPM1J | 1 | 113054138-113059473 | 5335 | 12 | 12 | 0.54 |
| PPP1R13L | 19 | 50574736-50601410 | 26674 | 3 | 4 | 0.14 |
| PPP1R16A | 8 | 145686224-145698312 | 12088 | 3 | 3 | 0.02 |
| PPP2R1B | 11 | 111102841-111142379 | 39538 | 23 | 18 | 0.26 |
| PPP2R5C | 14 | 101297951-101463830 | 165879 | 9 | 8 | 0.74 |
| PPP2R5D | 6 | 43060307-43088058 | 27751 | 2 | 3 | 0.02 |
| PQLC1 | 18 | 75763474-75812605 | 49131 | 10 | 11 | 0.18 |
| PRG3 | 11 | 56900818-56905199 | 4381 | 7 | 8 | 0.45 |
| PRMT5 | 14 | 22459572-22468501 | 8929 | 7 | 8 | 0.25 |
| PRPF40B | 12 | 48303619-48324716 | 21097 | 15 | 16 | 0.28 |
| PSAT1 | 9 | 80101878-80134829 | 32951 | 5 | 6 | 0.72 |
| PSEN1 | 14 | 72672931-72756862 | 83931 | 8 | 7 | 0.09 |
| PSMB4 | 1 | 149638664-149641036 | 2372 | 7 | 6 | 1.12 |
| PSMB8 | 6 | 32916471-32920690 | 4219 | 12 | 10 | 0.66 |
| PSMD2 | 3 | 185499715-185509534 | 9819 | 11 | 12 | 0.45 |
| PSMD7 | 16 | 72888181-72897687 | 9506 | 2 | 3 | 0.02 |
| PSME2 | 14 | 23682413-23685695 | 3282 | 5 | 7 | 0.92 |
| PSRC1 | 1 | 109623700-109627331 | 3631 | 19 | 20 | 0.69 |
| PTGER3 | 1 | 71090623-71286079 | 195456 | 7 | 6 | 0.32 |
| PTGFR | 1 | 78729315-78778974 | 49659 | 8 | 10 | 0.25 |
| PTPN9 | 15 | 73546514-73658680 | 112166 | 5 | 6 | 0.03 |
| PYGM | 11 | 64270436-64284763 | 14327 | 22 | 20 | 0.36 |
| QPCT | 2 | 37425256-37453969 | 28713 | 12 | 15 | 1.15 |
| R3HDM1 | 2 | 136005552-136199309 | 193757 | 14 | 14 | 0.52 |
| RAB36 | 22 | 21817512-21836531 | 19019 | 6 | 8 | 1.02 |
| RAB38 | 11 | 87486078-87548247 | 62169 | 7 | 7 | 0.66 |
| RAB40B | 17 | 78208237-78249802 | 41565 | 7 | 6 | 0.12 |
| RAD51AP1 | 12 | 4518316-4539475 | 21159 | 6 | 8 | 0.12 |
| RAD54B | 8 | 95453363-95556486 | 103123 | 24 | 23 | 1.22 |
| RALY | 20 | 32045392-32136336 | 90944 | 4 | 5 | 0.09 |
| RAN | 12 | 129922520-129927316 | 4796 | 4 | 5 | 0.06 |
| RARB | 3 | 25443023-25614424 | 171401 | 10 | 10 | 0.19 |
| RBPSUH | 4 | 25930430-26045851 | 115421 | 8 | 11 | 0.93 |
| RCL1 | 9 | 4782833-4851064 | 68231 | 14 | 15 | 0.13 |
| RCN2 | 15 | 75011170-75029300 | 18130 | 3 | 4 | 0.18 |
| RDH11 | 14 | 67213270-67232263 | 18993 | 5 | 6 | 0.1 |
| RDH12 | 14 | 67238355-67270921 | 32566 | 9 | 10 | 0.4 |
| RECQL5 | 17 | 71134544-71174864 | 40320 | 11 | 12 | 0.36 |
| REXO2 | 11 | 113815386-113826210 | 10824 | 4 | 5 | 0.09 |
| RFXANK | 19 | 19164007-19173678 | 9671 | 8 | 8 | 0.49 |
| RGS7 | 1 | 239005439-239587101 | 581662 | 8 | 9 | 0.11 |
| RHOH | 4 | 39874921-39922676 | 47755 | 4 | 5 | 0.05 |
| RHOT1 | 17 | 27493585-27576859 | 83274 | 9 | 10 | 0.8 |
| RIC8A | 11 | 198529-205110 | 6581 | 12 | 10 | 0.25 |
| RIOK1 | 6 | 7335060-7363269 | 28209 | 14 | 15 | 0.83 |
| RIPK1 | 6 | 3009120-3060420 | 51300 | 17 | 19 | 0.63 |
| RIPK3 | 14 | 23875066-23879082 | 4016 | 14 | 15 | 0.77 |
| RNASE6 | 14 | 20319049-20320466 | 1417 | 8 | 9 | 0.73 |
| RNF113B | 13 | 97626039-97627522 | 1483 | 10 | 13 | 0.77 |
| RNF122 | 8 | 33524814-33544185 | 19371 | 5 | 8 | 1.28 |
| RNF150 | 4 | 142006174-142274066 | 267892 | 8 | 8 | 0.23 |
| RNF25 | 2 | 219236830-219245025 | 8195 | 20 | 19 | 0.72 |
| RNF44 | 5 | 175886305-175897027 | 10722 | 5 | 6 | 0.06 |
| RNMT | 18 | 13716703-13754554 | 37851 | 13 | 13 | 0.17 |
| RPL27A | 11 | 8660570-8667995 | 7425 | 3 | 3 | 0.47 |
| RPS24 | 10 | 79463579-79485216 | 21637 | 4 | 5 | 0.05 |
| RRAS | 19 | 54830363-54835212 | 4849 | 4 | 5 | 0.57 |
| RUNX2 | 6 | 45404031-45626797 | 222766 | 7 | 8 | 0.08 |
| SAMD14 | 17 | 45545033-45562166 | 17133 | 7 | 7 | 0.06 |
| SART3 | 12 | 107440120-107479306 | 39186 | 20 | 22 | 1.83 |
| SCGB2A2 | 11 | 61794205-61797204 | 2999 | 3 | 4 | 0.08 |
| SDF2L1 | 22 | 20326541-20328588 | 2047 | 9 | 8 | 0.14 |
| SEC11C | 18 | 54958104-54977043 | 18939 | 4 | 4 | 0.08 |
| SEC13 | 3 | 10317614-10337855 | 20241 | 5 | 6 | 0.06 |
| SEC14L3 | 22 | 29185218-29198017 | 12799 | 20 | 17 | 1.58 |
| SERAC1 | 6 | 158450534-158509257 | 58723 | 17 | 14 | 0.91 |
| SERPINA11 | 14 | 93978553-93988875 | 10322 | 18 | 19 | 0.81 |
| SETD2 | 3 | 47032909-47180461 | 147552 | 37 | 39 | 1.6 |
| SETD8 | 12 | 122434656-122459853 | 25197 | 5 | 5 | 0.05 |
| SF3B14 | 2 | 24143957-24152818 | 8861 | 2 | 3 | 0.01 |
| SFRS1 | 17 | 53433278-53439706 | 6428 | 2 | 3 | 0.02 |
| SGCB | 4 | 52581628-52599203 | 17575 | 4 | 5 | 0.04 |
| SGK3 | 8 | 67742404-67936811 | 194407 | 4 | 5 | 0.46 |
| SGPL1 | 10 | 72245721-72309873 | 64152 | 10 | 11 | 0.9 |
| SIAH1 | 16 | 46947777-47039810 | 92033 | 2 | 3 | 0.22 |
| SIRT1 | 10 | 69314432-69348149 | 33717 | 13 | 14 | 0.57 |
| SKAP1 | 17 | 43565800-43862593 | 296793 | 7 | 9 | 1.06 |
| SLAMF8 | 1 | 158063102-158073906 | 10804 | 15 | 13 | 0.59 |
| SLC12A2 | 5 | 127447381-127553279 | 105898 | 13 | 12 | 0.75 |
| SLC16A10 | 6 | 111515501-111650907 | 135406 | 6 | 7 | 0.18 |
| SLC17A3 | 6 | 25953306-25982450 | 29144 | 11 | 13 | 1.3 |
| SLC25A31 | 4 | 128871023-128914897 | 43874 | 6 | 6 | 0.82 |
| SLC26A2 | 5 | 149320492-149347156 | 26664 | 23 | 24 | 0.72 |
| SLC30A1 | 1 | 209815003-209818722 | 3719 | 6 | 7 | 0.06 |
| SLC30A3 | 2 | 27330943-27339464 | 8521 | 3 | 3 | 0.31 |
| SLC34A2 | 4 | 25266532-25289466 | 22934 | 28 | 24 | 0.6 |
| SLC35B1 | 17 | 45133688-45140527 | 6839 | 4 | 5 | 0.21 |
| SLC3A2 | 11 | 62380093-62412929 | 32836 | 13 | 11 | 0.78 |
| SLC43A1 | 11 | 57008582-57039735 | 31153 | 11 | 11 | 0.27 |
| SLC44A2 | 19 | 10574132-10616232 | 42100 | 15 | 16 | 0.95 |
| SLC4A1AP | 2 | 27739841-27771351 | 31510 | 13 | 14 | 0.89 |
| SLCO1A2 | 12 | 21311650-21439638 | 127988 | 13 | 18 | 0.44 |
| SMC5 | 9 | 72063697-72159609 | 95912 | 12 | 12 | 1.23 |
| SNX5 | 20 | 17870243-17897490 | 27247 | 6 | 6 | 0.79 |
| SOCS4 | 14 | 54563593-54585959 | 22366 | 9 | 10 | 0.27 |
| SPINK2 | 4 | 57370790-57382650 | 11860 | 3 | 4 | 0.03 |
| SPP2 | 2 | 234624084-234650515 | 26431 | 6 | 8 | 0.85 |
| SPRR2G | 1 | 151388681-151390051 | 1370 | 3 | 4 | 0.09 |
| SPTLC2 | 14 | 77043022-77152863 | 109841 | 12 | 17 | 0.62 |
| SRGAP1 | 12 | 62524807-62823829 | 299022 | 22 | 27 | 1.13 |
| SRPR | 11 | 125638043-125643960 | 5917 | 16 | 15 | 0.68 |
| SS18L1 | 20 | 60152216-60190961 | 38745 | 9 | 9 | 0.23 |
| ST3GAL3 | 1 | 43945804-44169418 | 223614 | 6 | 7 | 0.59 |
| ST3GAL6 | 3 | 99933841-99995926 | 62085 | 5 | 7 | 0.24 |
| ST6GALNAC3 | 1 | 76312991-76869220 | 556229 | 11 | 13 | 0.85 |
| ST7L | 1 | 112867663-112963928 | 96265 | 18 | 19 | 0.78 |
| STK38L | 12 | 27288374-27369795 | 81421 | 3 | 4 | 0.16 |
| STOM | 9 | 123141173-123172366 | 31193 | 5 | 5 | 0.12 |
| STOML1 | 15 | 72062612-72074002 | 11390 | 7 | 6 | 0.68 |
| STX16 | 20 | 56659733-56687988 | 28255 | 8 | 4 | 0.9 |
| STXBP3 | 1 | 109090807-109153671 | 62864 | 5 | 6 | 0.77 |
| STXBP4 | 17 | 50401124-50596448 | 195324 | 16 | 17 | 1.08 |
| SUNC1 | 7 | 47993270-48035241 | 41971 | 11 | 10 | 0.14 |
| SUZ12 | 17 | 27288184-27352162 | 63978 | 3 | 4 | 0.07 |
| SYNGR1 | 22 | 38075899-38111539 | 35640 | 6 | 7 | 0.07 |
| SYNGR2 | 17 | 73676265-73680604 | 4339 | 5 | 6 | 0.46 |
| SYNJ1 | 21 | 32922943-33022148 | 99205 | 23 | 22 | 0.93 |
| SYTL2 | 11 | 85082912-85146692 | 63780 | 41 | 32 | 1.97 |
| TAF11 | 6 | 34953532-34963797 | 10265 | 4 | 5 | 0.11 |
| TARS2 | 1 | 148726543-148746373 | 19830 | 11 | 12 | 0.51 |
| TEX14 | 17 | 53989037-54124415 | 135378 | 41 | 37 | 1.9 |
| THOC6 | 16 | 3014042-3017752 | 3710 | 8 | 8 | 0.26 |
| THRB | 3 | 24133648-24511457 | 377809 | 9 | 10 | 0.39 |
| TIA1 | 2 | 70290079-70329283 | 39204 | 10 | 12 | 0.24 |
| TIAF1 | 17 | 24424664-24426753 | 2089 | 7 | 8 | 0.16 |
| TIMM8B | 11 | 111461079-111462669 | 1590 | 2 | 3 | 0.03 |
| TIPRL | 1 | 166414794-166437975 | 23181 | 2 | 2 | 0.02 |
| TKTL2 | 4 | 164611696-164614497 | 2801 | 21 | 19 | 1.03 |
| TLL1 | 4 | 167013859-167244443 | 230584 | 31 | 47 | 1.38 |
| TMCC1 | 3 | 130849324-131095093 | 245769 | 13 | 11 | 0.55 |
| TMEM101 | 17 | 39444081-39447871 | 3790 | 2 | 4 | 0.07 |
| TMEM106C | 12 | 46643633-46648928 | 5295 | 13 | 16 | 0.89 |
| TMEM133 | 11 | 100368020-100369876 | 1856 | 6 | 9 | 1.06 |
| TMEM177 | 2 | 120153212-120197661 | 44449 | 19 | 15 | 0.99 |
| TMEM182 | 2 | 102719825-102800570 | 80745 | 5 | 6 | 0.07 |
| TMEM63A | 1 | 224099862-224136671 | 36809 | 34 | 31 | 1.97 |
| TMEM67 | 8 | 94836247-94899523 | 63276 | 12 | 12 | 0.15 |
| TMOD2 | 15 | 49831101-49889635 | 58534 | 4 | 5 | 0.05 |
| TMPRSS11D | 4 | 68369188-68432311 | 63123 | 4 | 6 | 0.07 |
| TOP3B | 22 | 20641402-20667193 | 25791 | 17 | 19 | 0.56 |
| TOR1A | 9 | 131615041-131626262 | 11221 | 4 | 4 | 0.78 |
| TPM3 | 1 | 152394403-152431233 | 36830 | 2 | 3 | 0.01 |
| TPM4 | 19 | 16039347-16074408 | 35061 | 4 | 6 | 0.35 |
| TPSD1 | 16 | 1246273-1248495 | 2222 | 4 | 5 | 0.18 |
| TRAFD1 | 12 | 111047746-111075795 | 28049 | 11 | 13 | 0.68 |
| TRAPPC2L | 16 | 87450128-87456585 | 6457 | 7 | 4 | 0.17 |
| TRIM2 | 4 | 154293719-154479918 | 186199 | 16 | 17 | 1.12 |
| TRIM3 | 11 | 6426418-6452265 | 25847 | 10 | 8 | 0.52 |
| TRIM33 | 1 | 114736921-114855304 | 118383 | 17 | 16 | 1.28 |
| TRIM55 | 8 | 67201831-67250272 | 48441 | 2 | 3 | 0.15 |
| TRIM62 | 1 | 33383590-33419854 | 36264 | 6 | 7 | 0.32 |
| TRMT5 | 14 | 60507919-60517535 | 9616 | 19 | 17 | 0.35 |
| TRPC4 | 13 | 37108794-37341935 | 233141 | 17 | 20 | 0.7 |
| TSPAN3 | 15 | 75125385-75150568 | 25183 | 6 | 7 | 0.72 |
| TTC26 | 7 | 138469063-138525086 | 56023 | 9 | 8 | 0.38 |
| TTLL11 | 9 | 123624025-123895706 | 271681 | 9 | 10 | 0.15 |
| TTLL13 | 15 | 88593767-88603316 | 9549 | 8 | 15 | 0.96 |
| TWF2 | 3 | 52237665-52248223 | 10558 | 7 | 7 | 0.35 |
| TXNL1 | 18 | 52421050-52469500 | 48450 | 4 | 5 | 0.45 |
| U2AF1 | 21 | 43386134-43400757 | 14623 | 4 | 5 | 0.16 |
| UAP1 | 1 | 160797919-160836257 | 38338 | 9 | 11 | 0.36 |
| UBE2V1 | 20 | 48131067-48165901 | 34834 | 3 | 4 | 0.09 |
| UGT1A6 | 2 | 234265059-234346690 | 81631 | 20 | 16 | 1.1 |
| ULBP1 | 6 | 150326835-150336539 | 9704 | 5 | 5 | 0.08 |
| UNC50 | 2 | 98591473-98601409 | 9936 | 2 | 3 | 0.01 |
| UNQ9438 | 14 | 57932396-57945172 | 12776 | 2 | 3 | 0.02 |
| UPP1 | 7 | 48094879-48114855 | 19976 | 9 | 9 | 0.19 |
| VAMP1 | 12 | 6441664-6450104 | 8440 | 2 | 3 | 0.6 |
| VAMP3 | 1 | 7753915-7764079 | 10164 | 3 | 4 | 0.02 |
| VGF | 7 | 100592515-100595572 | 3057 | 6 | 7 | 0.08 |
| VIM | 10 | 17310475-17319598 | 9123 | 6 | 7 | 0.31 |
| VLDLR | 9 | 2611792-2644485 | 32693 | 23 | 19 | 1.2 |
| VN1R1 | 19 | 62658353-62659666 | 1313 | 5 | 6 | 0.72 |
| VNN3 | 6 | 133085618-133097596 | 11978 | 8 | 10 | 1.2 |
| VTCN1 | 1 | 117487731-117555072 | 67341 | 5 | 6 | 0.15 |
| VTI1B | 14 | 67187618-67211301 | 23683 | 8 | 9 | 0.57 |
| WDR8 | 1 | 3537197-3556497 | 19300 | 20 | 22 | 1 |
| WFDC3 | 20 | 43836253-43853954 | 17701 | 7 | 9 | 1.04 |
| WFDC5 | 20 | 43171506-43177217 | 5711 | 6 | 7 | 0.32 |
| WNT16 | 7 | 120752656-120768394 | 15738 | 12 | 12 | 1.03 |
| XRCC5 | 2 | 216682376-216779248 | 96872 | 10 | 11 | 0.47 |
| YOD1 | 1 | 205283816-205292948 | 9132 | 9 | 8 | 0.24 |
| ZBTB16 | 11 | 113435640-113626607 | 190967 | 10 | 11 | 0.18 |
| ZBTB26 | 9 | 124720198-124733600 | 13402 | 3 | 4 | 0.1 |
| ZDHHC6 | 10 | 114180047-114197296 | 17249 | 8 | 9 | 0.84 |
| ZFP2 | 5 | 178255521-178292816 | 37295 | 19 | 18 | 1.17 |
| ZFYVE21 | 14 | 103251897-103269752 | 17855 | 6 | 7 | 0.36 |
| ZKSCAN5 | 7 | 98940208-98969381 | 29173 | 19 | 11 | 0.35 |
| ZMPSTE24 | 1 | 40496319-40532443 | 36124 | 8 | 9 | 0.47 |
| ZMYM5 | 13 | 19295623-19335773 | 40150 | 11 | 9 | 0.46 |
| ZNF213 | 16 | 3125139-3132806 | 7667 | 11 | 10 | 0.5 |
| ZNF26 | 12 | 132073128-132099226 | 26098 | 10 | 8 | 1.03 |
| ZNF264 | 19 | 62394680-62426026 | 31346 | 21 | 18 | 1.07 |
| ZNF32 | 10 | 43459312-43464332 | 5020 | 6 | 7 | 0.15 |
| ZNF329 | 19 | 63329430-63358289 | 28859 | 14 | 13 | 0.26 |
| ZNF396 | 18 | 31200658-31211299 | 10641 | 8 | 9 | 0.24 |
| ZNF474 | 5 | 121493113-121517165 | 24052 | 27 | 25 | 1.2 |
| ZNF521 | 18 | 20895888-21186114 | 290226 | 27 | 21 | 0.95 |
| ZNF555 | 19 | 2792481-2805207 | 12726 | 12 | 11 | 0.53 |
| ZNF571 | 19 | 42737523-42777513 | 39990 | 21 | 25 | 1.36 |
| ZNF576 | 19 | 48792383-48795996 | 3613 | 5 | 6 | 0.24 |
| ZNF583 | 19 | 61607529-61628212 | 20683 | 5 | 6 | 0.65 |
| ZNF616 | 19 | 57309464-57335003 | 25539 | 23 | 22 | 1.28 |
| ZNF625 | 19 | 12111994-12128529 | 16535 | 5 | 5 | 1.08 |
| ZNF642 | 1 | 40715888-40734602 | 18714 | 14 | 16 | 0.39 |
| ZNF689 | 16 | 30522200-30529183 | 6983 | 8 | 8 | 0.42 |
| ZNHIT4 | 2 | 74535706-74538593 | 2887 | 3 | 4 | 0.02 |
| ZPBP2 | 17 | 35277980-35287675 | 9695 | 18 | 16 | 1.42 |
| ZSCAN2 | 15 | 82945252-82967951 | 22699 | 2 | 3 | 0.02 |
| ZSWIM1 | 20 | 43943286-43947312 | 4026 | 9 | 9 | 0.44 |
