# Supplementary material for: Sequencing genes in silico using single nucleotide polymorphisms
Source: BMC Genet. 2012 Jan 30;13:6. doi: 10.1186/1471-2156-13-6 (PMC3283449; doi:10.1186/1471-2156-13-6)
Supplement: Additional file 3 — Supplement Table 2. Accuracy % (call rate %) of the prediction models evaluated on training and validation set. [file 1471-2156-13-6-S3.DOCX]

| **Supplement Table 2: Accuracy % (call rate %) of the predictive models evaluated on training set and the validation set** | | | | | | |
| --- | --- | --- | --- | --- | --- | --- |
|  |  |  |  |  |  |  |
|  | **Training Set** | | | **Validation Set** | | |
| **gene** | **CT=0** | **CT=0.5** | **CT=0.9** | **CT=0** | **CT=0.5** | **CT=0.9** |
| AASDHPPT | 100 | 100(100) | 100(97) | 99 | 99(100) | 99(97) |
| AATF | 99 | 99(100) | 100(94) | 94 | 94(100) | 94(93) |
| ABCB9 | 98 | 98(99) | 100(80) | 97 | 97(100) | 98(72) |
| ABLIM3 | 98 | 99(100) | 99(93) | 93 | 94(97) | 95(90) |
| ABTB2 | 98 | 98(100) | 99(90) | 90 | 90(98) | 93(88) |
| ACN9 | 99 | 99(100) | 99(97) | 98 | 98(100) | 99(95) |
| ACOX3 | 96 | 96(100) | 100(67) | 90 | 91(98) | 93(59) |
| ACPL2 | 98 | 98(99) | 99(87) | 96 | 97(97) | 99(86) |
| ACTL7B | 97 | 97(100) | 98(90) | 94 | 94(99) | 95(90) |
| ADAM29 | 97 | 97(99) | 99(80) | 88 | 89(97) | 91(74) |
| ADD2 | 99 | 99(99) | 100(96) | 99 | 99(99) | 99(91) |
| AGPAT3 | 99 | 99(100) | 99(96) | 95 | 95(99) | 97(94) |
| AGPAT4 | 99 | 99(100) | 99(93) | 97 | 97(100) | 98(95) |
| AHSA2 | 100 | 100(100) | 100(99) | 99 | 99(99) | 99(98) |
| AIF1 | 99 | 99(100) | 99(99) | 99 | 99(100) | 99(99) |
| AIG1 | 99 | 99(100) | 99(95) | 96 | 96(100) | 96(96) |
| AK7 | 97 | 97(99) | 99(81) | 89 | 89(98) | 93(71) |
| AKAP7 | 98 | 98(100) | 99(91) | 96 | 96(100) | 96(89) |
| AKR1B1 | 99 | 99(100) | 99(89) | 94 | 95(99) | 97(89) |
| AKR1CL1 | 98 | 98(100) | 99(89) | 96 | 96(100) | 96(86) |
| AKR1D1 | 99 | 99(100) | 100(91) | 98 | 98(100) | 99(93) |
| ALDH1A2 | 98 | 98(100) | 99(95) | 97 | 97(100) | 98(94) |
| ALS2CR13 | 100 | 100(100) | 100(97) | 99 | 99(100) | 99(98) |
| AMPD2 | 99 | 99(100) | 100(92) | 96 | 96(100) | 97(93) |
| ANAPC5 | 97 | 98(97) | 99(87) | 92 | 93(96) | 95(82) |
| ANKRD12 | 98 | 98(100) | 99(86) | 95 | 95(99) | 96(81) |
| ANP32C | 97 | 97(100) | 97(85) | 92 | 93(97) | 94(80) |
| APCDD1 | 97 | 97(100) | 98(76) | 88 | 89(94) | 90(75) |
| APITD1 | 100 | 100(100) | 100(99) | 100 | 100(100) | 100(100) |
| APOBEC3F | 97 | 97(100) | 99(82) | 95 | 96(99) | 97(80) |
| AQP3 | 98 | 98(100) | 99(96) | 97 | 97(100) | 97(93) |
| ARL6IP6 | 100 | 100(100) | 100(99) | 99 | 99(100) | 100(99) |
| ARNT | 98 | 98(100) | 99(93) | 97 | 97(100) | 97(91) |
| ASAH2 | 97 | 97(99) | 98(88) | 95 | 95(99) | 96(86) |
| ASL | 99 | 99(99) | 100(93) | 99 | 99(100) | 100(92) |
| ATG5 | 99 | 99(99) | 100(94) | 98 | 98(100) | 99(96) |
| ATHL1 | 93 | 93(95) | 100(58) | 83 | 84(88) | 86(49) |
| ATP2B1 | 97 | 97(100) | 99(71) | 97 | 97(100) | 100(73) |
| ATP2C1 | 99 | 99(100) | 99(97) | 97 | 97(100) | 99(92) |
| ATRNL1 | 98 | 98(100) | 99(91) | 92 | 92(99) | 94(79) |
| B3GALTL | 99 | 99(100) | 99(95) | 97 | 97(99) | 97(94) |
| B4GALT6 | 99 | 99(100) | 100(95) | 98 | 98(98) | 99(95) |
| BAG2 | 100 | 100(99) | 100(99) | 100 | 100(99) | 100(99) |
| BAZ1A | 98 | 98(100) | 99(91) | 96 | 96(99) | 96(89) |
| BCAP29 | 100 | 100(100) | 100(100) | 99 | 99(100) | 99(100) |
| BCHE | 98 | 98(100) | 99(83) | 95 | 95(100) | 97(78) |
| BCL11A | 92 | 93(97) | 98(70) | 93 | 93(98) | 97(69) |
| BCL2L11 | 99 | 99(99) | 100(91) | 99 | 99(100) | 99(92) |
| BDH1 | 97 | 98(99) | 99(84) | 91 | 92(95) | 94(83) |
| BLMH | 99 | 99(100) | 99(97) | 99 | 99(100) | 99(98) |
| BOLL | 100 | 100(99) | 100(98) | 100 | 100(100) | 100(99) |
| BRD7 | 99 | 99(100) | 99(98) | 96 | 96(100) | 96(96) |
| BRMS1L | 100 | 100(100) | 100(98) | 100 | 100(100) | 100(98) |
| BTBD1 | 99 | 99(100) | 100(96) | 99 | 99(100) | 99(98) |
| BXDC2 | 100 | 100(100) | 100(98) | 97 | 97(100) | 98(96) |
| C10orf65 | 93 | 94(97) | 98(45) | 84 | 85(96) | 89(47) |
| C10orf78 | 99 | 99(100) | 99(100) | 99 | 99(100) | 99(99) |
| C11orf53 | 97 | 97(99) | 98(83) | 96 | 96(99) | 97(80) |
| C12orf63 | 95 | 95(99) | 97(63) | 90 | 90(98) | 90(53) |
| C14orf1 | 99 | 99(100) | 100(96) | 99 | 99(100) | 99(97) |
| C14orf108 | 99 | 99(100) | 100(93) | 100 | 100(100) | 100(93) |
| C15orf40 | 99 | 99(100) | 99(95) | 99 | 99(100) | 99(96) |
| C16orf48 | 99 | 99(100) | 100(93) | 98 | 98(100) | 99(92) |
| C16orf55 | 99 | 99(100) | 100(96) | 97 | 97(100) | 97(96) |
| C17orf25 | 98 | 98(100) | 99(86) | 93 | 93(100) | 95(89) |
| C17orf53 | 97 | 98(99) | 99(88) | 97 | 97(100) | 98(84) |
| C17orf66 | 97 | 98(98) | 98(89) | 93 | 94(96) | 95(85) |
| C17orf79 | 99 | 99(100) | 99(95) | 98 | 98(100) | 99(94) |
| C18orf10 | 99 | 99(100) | 99(98) | 98 | 98(98) | 99(96) |
| C19orf26 | 97 | 97(99) | 99(84) | 98 | 98(99) | 99(80) |
| C1orf178 | 99 | 99(100) | 100(91) | 98 | 98(100) | 98(90) |
| C1orf198 | 99 | 99(99) | 100(93) | 96 | 96(100) | 98(93) |
| C1orf63 | 98 | 98(100) | 99(93) | 96 | 97(99) | 98(91) |
| C1orf9 | 96 | 96(100) | 98(81) | 93 | 93(99) | 94(82) |
| C1orf94 | 96 | 96(97) | 98(83) | 89 | 89(96) | 92(81) |
| C1QBP | 100 | 100(100) | 100(99) | 99 | 99(100) | 99(99) |
| C2orf18 | 99 | 99(100) | 100(90) | 98 | 98(100) | 99(94) |
| C2orf47 | 99 | 99(100) | 100(94) | 99 | 99(100) | 100(94) |
| C3orf14 | 100 | 100(99) | 100(95) | 98 | 98(100) | 99(93) |
| C3orf37 | 99 | 99(100) | 99(94) | 98 | 99(99) | 99(91) |
| C3orf38 | 98 | 98(100) | 99(91) | 98 | 98(100) | 98(93) |
| C4orf33 | 96 | 96(100) | 98(83) | 97 | 97(100) | 98(85) |
| C5orf25 | 98 | 98(100) | 98(97) | 98 | 98(100) | 98(94) |
| C6orf117 | 98 | 98(100) | 99(96) | 99 | 99(100) | 99(97) |
| C6orf130 | 100 | 100(100) | 100(99) | 99 | 99(100) | 99(98) |
| C6orf145 | 98 | 98(100) | 99(95) | 97 | 97(98) | 97(89) |
| C6orf51 | 100 | 100(100) | 100(98) | 100 | 100(100) | 100(98) |
| C9orf131 | 95 | 95(100) | 99(65) | 91 | 92(98) | 96(59) |
| CALML5 | 96 | 96(100) | 99(64) | 91 | 91(98) | 95(62) |
| CAPNS1 | 100 | 100(100) | 100(100) | 99 | 99(100) | 99(100) |
| CART1 | 98 | 98(100) | 99(90) | 96 | 96(100) | 98(87) |
| CASP10 | 99 | 99(100) | 100(92) | 95 | 95(100) | 96(91) |
| CASP7 | 99 | 99(100) | 100(90) | 95 | 95(98) | 95(89) |
| CBLB | 97 | 97(98) | 99(80) | 89 | 90(96) | 93(66) |
| CCBE1 | 99 | 99(100) | 99(95) | 98 | 98(100) | 98(95) |
| CCDC63 | 98 | 98(100) | 99(84) | 90 | 92(97) | 94(75) |
| CCK | 100 | 100(100) | 100(100) | 99 | 99(100) | 99(100) |
| CCKAR | 98 | 98(100) | 100(91) | 94 | 94(100) | 96(88) |
| CCNDBP1 | 99 | 99(100) | 99(98) | 99 | 99(100) | 99(97) |
| CCT6B | 97 | 98(97) | 99(76) | 91 | 93(93) | 93(69) |
| CD1C | 97 | 97(100) | 99(85) | 96 | 96(99) | 98(89) |
| CD300LB | 98 | 98(100) | 98(93) | 98 | 98(100) | 98(93) |
| CD46 | 99 | 99(100) | 100(98) | 100 | 100(100) | 100(98) |
| CD79B | 98 | 98(100) | 99(89) | 97 | 97(100) | 98(87) |
| CD9 | 100 | 100(100) | 100(98) | 99 | 99(100) | 99(99) |
| CDC25C | 98 | 98(100) | 100(88) | 99 | 99(100) | 99(87) |
| CDCA2 | 96 | 96(99) | 98(68) | 90 | 92(96) | 92(61) |
| CDKN2A | 100 | 100(100) | 100(99) | 100 | 100(100) | 100(99) |
| CENPH | 100 | 100(100) | 100(99) | 100 | 100(100) | 100(100) |
| CEP250 | 95 | 96(98) | 100(71) | 89 | 91(96) | 91(65) |
| CETN1 | 93 | 93(98) | 98(42) | 86 | 87(97) | 88(40) |
| CHEK1 | 99 | 99(100) | 100(98) | 99 | 99(100) | 99(98) |
| CHFR | 97 | 97(100) | 98(93) | 96 | 96(100) | 98(92) |
| CIDEA | 96 | 96(100) | 97(82) | 96 | 97(99) | 97(83) |
| CLDN10 | 100 | 100(100) | 100(98) | 99 | 99(100) | 99(98) |
| CLDN19 | 100 | 100(100) | 100(99) | 99 | 99(100) | 99(99) |
| CLEC2D | 99 | 99(100) | 99(97) | 98 | 98(100) | 99(98) |
| CLRN3 | 97 | 97(100) | 99(87) | 97 | 97(100) | 98(86) |
| CLSPN | 96 | 97(98) | 99(79) | 91 | 90(98) | 96(73) |
| CMPK | 100 | 100(100) | 100(100) | 99 | 99(100) | 99(99) |
| CNTFR | 100 | 100(100) | 99(100) | 98 | 98(100) | 98(99) |
| COG7 | 98 | 98(100) | 99(96) | 96 | 96(98) | 96(89) |
| COL4A3BP | 99 | 99(100) | 99(99) | 99 | 99(100) | 99(99) |
| COLQ | 99 | 99(100) | 99(96) | 98 | 98(100) | 99(94) |
| COMMD9 | 100 | 100(100) | 100(100) | 100 | 100(100) | 100(100) |
| COPS8 | 100 | 100(100) | 100(99) | 99 | 99(100) | 99(98) |
| CRB1 | 95 | 95(100) | 98(74) | 93 | 93(100) | 96(72) |
| CREB3L2 | 98 | 98(100) | 100(85) | 94 | 94(98) | 96(80) |
| CREB5 | 99 | 99(100) | 100(97) | 98 | 98(100) | 99(93) |
| CRELD1 | 97 | 97(100) | 99(86) | 90 | 91(100) | 94(86) |
| CRNN | 98 | 98(100) | 99(91) | 98 | 98(100) | 97(88) |
| CRYGA | 99 | 99(100) | 99(97) | 98 | 98(100) | 99(96) |
| CTDSPL | 99 | 99(100) | 100(91) | 99 | 99(100) | 99(90) |
| CTSS | 99 | 99(100) | 99(99) | 97 | 97(100) | 97(100) |
| CXCL13 | 100 | 100(100) | 100(100) | 99 | 99(100) | 99(100) |
| CYB561D1 | 99 | 99(100) | 100(96) | 97 | 97(100) | 99(95) |
| CYBASC3 | 100 | 100(100) | 100(98) | 99 | 99(100) | 99(100) |
| CYP2A13 | 99 | 100(99) | 100(96) | 99 | 100(99) | 100(97) |
| CYP2C18 | 98 | 98(100) | 98(84) | 95 | 95(100) | 96(85) |
| CYP3A7 | 98 | 98(100) | 99(89) | 97 | 97(100) | 97(93) |
| CYP4X1 | 100 | 100(100) | 100(98) | 99 | 99(100) | 99(99) |
| CYP7B1 | 99 | 99(100) | 99(91) | 98 | 98(100) | 99(94) |
| DAZAP2 | 99 | 99(100) | 100(97) | 99 | 99(100) | 100(97) |
| DCLRE1C | 90 | 93(89) | 97(55) | 82 | 85(83) | 85(47) |
| DCTN5 | 99 | 99(100) | 99(95) | 99 | 99(100) | 99(96) |
| DDI1 | 98 | 98(99) | 99(95) | 92 | 92(99) | 93(94) |
| DDX49 | 97 | 97(100) | 99(80) | 97 | 97(98) | 98(85) |
| DDX50 | 98 | 98(100) | 99(90) | 97 | 97(100) | 98(87) |
| DGKZ | 100 | 100(100) | 100(100) | 100 | 100(100) | 100(100) |
| DHRS7B | 99 | 99(100) | 99(96) | 98 | 98(100) | 99(95) |
| DKK4 | 99 | 99(100) | 99(98) | 98 | 98(100) | 98(95) |
| DLGAP4 | 97 | 97(100) | 99(81) | 96 | 97(99) | 98(80) |
| DNAJC15 | 99 | 99(100) | 99(98) | 98 | 98(100) | 97(95) |
| DUSP10 | 99 | 99(100) | 99(99) | 99 | 99(100) | 99(100) |
| DUSP26 | 99 | 99(100) | 100(83) | 96 | 96(100) | 97(77) |
| DYX1C1 | 100 | 100(100) | 100(99) | 98 | 98(100) | 98(98) |
| EFCAB3 | 98 | 98(100) | 99(93) | 97 | 97(100) | 98(92) |
| ELAVL4 | 99 | 99(100) | 99(92) | 92 | 92(100) | 93(85) |
| EMCN | 98 | 98(100) | 100(89) | 97 | 97(99) | 98(90) |
| ENDOG | 100 | 100(100) | 100(99) | 98 | 98(100) | 98(99) |
| ENTHD1 | 98 | 98(100) | 100(86) | 96 | 96(100) | 97(87) |
| EPB42 | 98 | 98(100) | 99(88) | 96 | 96(100) | 96(85) |
| EPN2 | 99 | 99(100) | 99(95) | 96 | 96(100) | 97(94) |
| EPYC | 99 | 99(100) | 99(93) | 97 | 97(100) | 98(88) |
| ERCC3 | 99 | 99(100) | 99(98) | 99 | 99(100) | 99(96) |
| ESAM | 99 | 99(100) | 99(91) | 94 | 94(98) | 97(81) |
| ETS2 | 98 | 98(99) | 99(79) | 92 | 92(98) | 94(64) |
| ETV5 | 99 | 99(100) | 100(94) | 98 | 98(100) | 99(92) |
| EXTL1 | 95 | 96(97) | 99(62) | 86 | 88(93) | 88(59) |
| F2RL1 | 98 | 98(100) | 98(93) | 98 | 98(99) | 99(91) |
| FADS3 | 99 | 99(100) | 100(93) | 99 | 99(100) | 100(95) |
| FAM105A | 97 | 98(99) | 100(82) | 97 | 97(98) | 98(84) |
| FAM112B | 100 | 100(100) | 100(94) | 99 | 99(100) | 100(97) |
| FAM117A | 99 | 99(100) | 100(92) | 96 | 96(100) | 98(88) |
| FAM19A3 | 97 | 97(99) | 99(88) | 97 | 97(100) | 97(85) |
| FAM19A4 | 100 | 100(100) | 100(98) | 99 | 99(100) | 99(98) |
| FAM73A | 99 | 99(100) | 99(97) | 99 | 99(100) | 99(95) |
| FAS | 99 | 99(99) | 100(97) | 97 | 98(97) | 99(94) |
| FASTKD5 | 95 | 98(88) | 99(79) | 91 | 97(85) | 97(73) |
| FBXL4 | 99 | 99(100) | 99(93) | 99 | 99(100) | 99(99) |
| FBXO24 | 99 | 99(100) | 100(97) | 99 | 99(100) | 99(96) |
| FCF1 | 98 | 98(99) | 99(88) | 98 | 98(99) | 99(86) |
| FGF3 | 98 | 98(100) | 100(91) | 96 | 96(99) | 98(90) |
| FIBP | 100 | 100(100) | 100(96) | 99 | 99(100) | 99(98) |
| FLJ10781 | 98 | 98(100) | 99(83) | 85 | 86(96) | 87(70) |
| FLJ16793 | 100 | 100(100) | 100(100) | 98 | 98(100) | 98(100) |
| FLJ31951 | 100 | 100(100) | 100(99) | 99 | 99(100) | 99(99) |
| FLJ46688 | 98 | 98(100) | 98(93) | 96 | 96(100) | 96(88) |
| FOXI1 | 98 | 98(100) | 98(98) | 95 | 95(100) | 95(96) |
| FSHR | 98 | 98(100) | 100(87) | 97 | 97(100) | 97(89) |
| FTSJ3 | 97 | 97(99) | 98(90) | 93 | 93(99) | 94(91) |
| FXC1 | 99 | 99(100) | 100(96) | 96 | 96(99) | 96(91) |
| FZD9 | 99 | 99(100) | 99(100) | 98 | 98(100) | 98(99) |
| GAB2 | 94 | 95(98) | 99(58) | 89 | 90(93) | 92(56) |
| GABPA | 97 | 97(100) | 99(78) | 96 | 96(100) | 99(82) |
| GALM | 97 | 97(100) | 99(87) | 96 | 96(99) | 98(83) |
| GALNT13 | 99 | 99(99) | 100(95) | 96 | 96(99) | 98(87) |
| GATAD2A | 97 | 97(99) | 100(78) | 94 | 94(97) | 96(69) |
| GBP6 | 94 | 95(99) | 98(63) | 90 | 91(96) | 95(57) |
| GDNF | 100 | 100(100) | 100(98) | 100 | 100(100) | 100(96) |
| GEFT | 96 | 96(100) | 98(87) | 93 | 94(98) | 96(81) |
| GENX-3414 | 100 | 100(100) | 100(95) | 99 | 99(100) | 100(91) |
| GGH | 99 | 99(100) | 100(97) | 99 | 99(100) | 99(96) |
| GIMAP7 | 95 | 95(99) | 98(72) | 95 | 96(97) | 97(71) |
| GJA5 | 99 | 99(100) | 99(97) | 98 | 98(100) | 99(93) |
| GLOXD1 | 100 | 100(100) | 100(99) | 98 | 98(100) | 98(99) |
| GLS2 | 98 | 98(99) | 100(85) | 95 | 96(98) | 97(84) |
| GMCL1 | 99 | 99(100) | 99(93) | 99 | 99(99) | 100(89) |
| GNG4 | 98 | 98(100) | 98(98) | 97 | 98(98) | 99(94) |
| GOPC | 99 | 99(100) | 99(99) | 99 | 99(100) | 99(99) |
| GPD2 | 97 | 97(99) | 98(76) | 94 | 96(97) | 98(75) |
| GPR114 | 96 | 97(98) | 99(83) | 89 | 90(97) | 91(84) |
| GPR120 | 99 | 99(100) | 100(89) | 97 | 97(100) | 98(86) |
| GPR128 | 98 | 98(100) | 99(90) | 93 | 93(99) | 95(82) |
| GPR37L1 | 97 | 97(100) | 98(81) | 91 | 91(100) | 92(84) |
| GPT2 | 99 | 99(100) | 99(96) | 95 | 95(100) | 96(94) |
| GPX7 | 99 | 99(100) | 99(95) | 96 | 96(100) | 97(93) |
| GRIA4 | 98 | 99(99) | 99(91) | 96 | 96(100) | 97(88) |
| GTDC1 | 98 | 98(100) | 99(87) | 95 | 95(99) | 97(79) |
| GTF2E2 | 99 | 99(100) | 100(96) | 99 | 99(100) | 100(96) |
| GTF2H5 | 100 | 100(100) | 100(100) | 100 | 100(100) | 100(100) |
| GUF1 | 96 | 96(100) | 98(81) | 98 | 98(100) | 99(84) |
| H2AFY2 | 97 | 97(100) | 100(81) | 97 | 97(99) | 98(81) |
| HAGH | 99 | 99(100) | 99(92) | 95 | 95(100) | 95(89) |
| HAX1 | 100 | 100(100) | 100(99) | 100 | 100(100) | 100(100) |
| HFE | 100 | 100(100) | 100(99) | 99 | 99(100) | 99(99) |
| HIF3A | 98 | 98(100) | 99(80) | 97 | 97(100) | 98(86) |
| HIGD2A | 100 | 100(100) | 100(95) | 99 | 99(100) | 99(94) |
| HINT3 | 99 | 99(100) | 99(97) | 98 | 98(100) | 99(96) |
| HIPK1 | 97 | 97(100) | 99(78) | 95 | 95(99) | 97(78) |
| HIRA | 99 | 99(99) | 100(91) | 97 | 97(100) | 97(89) |
| HISPPD1 | 96 | 96(100) | 97(72) | 91 | 91(98) | 92(74) |
| HIST1H2BC | 100 | 100(100) | 100(99) | 100 | 100(100) | 100(98) |
| HIST1H2BD | 98 | 98(100) | 100(96) | 97 | 98(100) | 99(94) |
| HIST1H2BJ | 99 | 99(100) | 100(97) | 97 | 97(100) | 98(95) |
| HIST1H2BO | 100 | 100(100) | 100(100) | 100 | 100(100) | 100(100) |
| HMGN4 | 99 | 99(100) | 99(100) | 98 | 98(100) | 98(99) |
| HNRPH1 | 98 | 98(100) | 99(87) | 98 | 98(100) | 98(89) |
| HNRPK | 99 | 99(100) | 99(99) | 99 | 99(100) | 99(100) |
| HRH4 | 99 | 99(100) | 99(96) | 95 | 95(99) | 96(92) |
| HSCB | 99 | 99(100) | 99(95) | 100 | 100(100) | 100(94) |
| HSD17B3 | 97 | 98(100) | 99(88) | 95 | 95(100) | 98(82) |
| HSD3B2 | 99 | 99(100) | 99(97) | 99 | 99(100) | 99(95) |
| HSP90AA1 | 97 | 97(100) | 98(90) | 92 | 93(98) | 94(83) |
| HSPA14 | 100 | 100(100) | 100(99) | 98 | 98(100) | 98(99) |
| HTATIP | 99 | 99(100) | 100(93) | 97 | 97(100) | 98(92) |
| HTR7 | 100 | 100(100) | 100(100) | 100 | 100(100) | 100(100) |
| ICAM4 | 99 | 99(100) | 100(96) | 99 | 99(99) | 99(97) |
| IFI44 | 98 | 98(100) | 99(87) | 94 | 94(100) | 96(84) |
| IFNGR2 | 98 | 98(100) | 99(97) | 98 | 98(100) | 98(95) |
| IKZF3 | 100 | 100(100) | 100(100) | 96 | 96(100) | 96(100) |
| IL15 | 100 | 100(100) | 100(99) | 99 | 99(100) | 99(100) |
| IL24 | 98 | 98(100) | 99(89) | 98 | 98(99) | 99(86) |
| IMP3 | 99 | 99(100) | 99(92) | 98 | 98(100) | 99(87) |
| IMPACT | 99 | 99(100) | 99(95) | 99 | 99(100) | 99(97) |
| INSIG1 | 99 | 99(100) | 100(95) | 98 | 98(100) | 99(96) |
| INVS | 97 | 97(100) | 98(78) | 94 | 94(100) | 94(79) |
| IRAK4 | 99 | 99(100) | 99(97) | 99 | 99(100) | 99(96) |
| IRF4 | 97 | 98(99) | 99(82) | 95 | 95(98) | 98(81) |
| IRF6 | 98 | 98(100) | 100(79) | 96 | 96(99) | 96(74) |
| ITGB4BP | 99 | 99(100) | 100(97) | 99 | 99(100) | 100(94) |
| JOSD1 | 99 | 99(100) | 99(95) | 98 | 98(100) | 98(94) |
| KCNJ11 | 96 | 96(98) | 99(73) | 87 | 87(97) | 89(70) |
| KCNK12 | 100 | 100(100) | 100(99) | 99 | 99(100) | 100(99) |
| KCNK13 | 97 | 97(99) | 98(83) | 92 | 93(99) | 95(72) |
| KIAA0737 | 97 | 97(100) | 98(91) | 96 | 96(100) | 97(93) |
| KIAA1446 | 99 | 99(100) | 100(94) | 98 | 98(100) | 98(94) |
| KIAA1622 | 98 | 98(100) | 100(83) | 94 | 94(99) | 97(76) |
| KIF9 | 95 | 95(99) | 98(58) | 93 | 93(100) | 94(59) |
| KLHL12 | 100 | 100(100) | 100(99) | 99 | 99(100) | 100(99) |
| KLHL31 | 96 | 97(99) | 99(73) | 92 | 92(99) | 93(72) |
| KRR1 | 98 | 98(100) | 99(89) | 97 | 97(100) | 97(86) |
| KRT18 | 100 | 100(100) | 100(100) | 100 | 100(100) | 100(100) |
| KRT27 | 92 | 94(93) | 98(54) | 86 | 89(93) | 91(54) |
| LASS3 | 98 | 99(99) | 100(94) | 94 | 94(100) | 97(92) |
| LASS4 | 98 | 98(100) | 99(89) | 97 | 97(100) | 97(91) |
| LCE1F | 98 | 98(100) | 100(84) | 94 | 94(100) | 98(83) |
| LCN10 | 98 | 99(100) | 99(95) | 98 | 99(97) | 98(92) |
| LEO1 | 99 | 99(100) | 99(97) | 96 | 96(100) | 96(96) |
| LEPREL1 | 98 | 98(98) | 99(94) | 89 | 90(95) | 90(90) |
| LGTN | 98 | 98(100) | 99(93) | 95 | 95(99) | 97(89) |
| LHX6 | 99 | 99(100) | 99(99) | 98 | 98(100) | 98(99) |
| LIMK1 | 97 | 97(99) | 99(81) | 94 | 94(100) | 97(72) |
| LIMK2 | 98 | 98(100) | 99(91) | 96 | 97(98) | 97(90) |
| LIPL1 | 99 | 99(100) | 99(98) | 96 | 96(100) | 96(98) |
| LOC133308 | 99 | 99(100) | 99(94) | 96 | 96(100) | 97(93) |
| LOC144097 | 98 | 98(100) | 99(92) | 98 | 98(100) | 98(92) |
| LOC153222 | 99 | 99(100) | 99(94) | 99 | 99(100) | 99(91) |
| LOC284402 | 98 | 98(100) | 100(94) | 97 | 97(100) | 98(94) |
| LOC389541 | 98 | 98(100) | 98(96) | 98 | 98(100) | 98(92) |
| LONRF2 | 97 | 97(98) | 98(87) | 92 | 92(99) | 93(85) |
| LPL | 96 | 96(99) | 99(79) | 88 | 90(95) | 94(66) |
| LRG1 | 97 | 97(100) | 98(77) | 92 | 93(97) | 96(70) |
| LRMP | 98 | 98(100) | 99(89) | 97 | 97(100) | 97(90) |
| LRRC20 | 100 | 100(99) | 100(97) | 99 | 99(100) | 99(98) |
| LRRC23 | 97 | 97(100) | 99(82) | 96 | 96(99) | 97(85) |
| LRRC52 | 97 | 98(99) | 99(83) | 95 | 96(98) | 95(82) |
| LTB4DH | 97 | 97(100) | 99(80) | 97 | 97(99) | 97(83) |
| LYPD2 | 100 | 100(100) | 100(99) | 99 | 99(100) | 99(99) |
| LYPD6 | 99 | 99(100) | 100(94) | 98 | 98(100) | 98(93) |
| MAP3K12 | 99 | 99(100) | 100(96) | 98 | 98(100) | 98(96) |
| MAPK9 | 100 | 100(100) | 100(100) | 98 | 98(100) | 98(99) |
| MASP1 | 98 | 98(100) | 98(92) | 90 | 91(92) | 93(77) |
| MBD1 | 98 | 98(100) | 99(88) | 97 | 97(99) | 96(87) |
| MCART1 | 98 | 98(100) | 100(94) | 99 | 99(100) | 99(96) |
| MCMDC1 | 98 | 98(100) | 99(91) | 96 | 96(100) | 96(91) |
| MELK | 94 | 94(100) | 98(59) | 92 | 92(93) | 93(59) |
| MFAP1 | 98 | 98(100) | 99(89) | 98 | 98(100) | 98(84) |
| MFSD2 | 97 | 97(100) | 98(85) | 97 | 97(99) | 98(81) |
| MGC14376 | 100 | 100(100) | 100(99) | 99 | 99(100) | 100(99) |
| MGC70857 | 100 | 100(100) | 100(100) | 99 | 99(100) | 99(99) |
| MKL2 | 98 | 98(100) | 99(96) | 97 | 97(100) | 97(98) |
| MOV10 | 94 | 94(99) | 98(73) | 93 | 93(98) | 97(71) |
| MRCL3 | 100 | 100(100) | 100(99) | 99 | 99(100) | 99(97) |
| MRPL39 | 98 | 98(100) | 99(91) | 95 | 95(99) | 95(92) |
| MRPS18B | 99 | 99(100) | 100(92) | 99 | 99(100) | 99(92) |
| MRPS7 | 99 | 99(100) | 99(98) | 99 | 99(100) | 99(97) |
| MSH4 | 97 | 98(99) | 99(75) | 94 | 95(99) | 96(70) |
| MTERFD2 | 99 | 99(100) | 99(95) | 97 | 97(100) | 97(95) |
| MTMR2 | 98 | 98(100) | 99(87) | 93 | 93(100) | 95(80) |
| MTP18 | 97 | 97(100) | 99(79) | 97 | 97(100) | 96(75) |
| MYB | 99 | 99(100) | 100(92) | 95 | 95(100) | 96(90) |
| MYEOV2 | 98 | 98(99) | 98(88) | 98 | 98(100) | 98(92) |
| NAGLU | 98 | 98(100) | 99(96) | 95 | 95(100) | 96(90) |
| NCKIPSD | 99 | 99(100) | 99(91) | 99 | 99(100) | 100(90) |
| NCSTN | 95 | 95(99) | 98(72) | 94 | 95(97) | 98(72) |
| NEK7 | 99 | 99(100) | 100(88) | 99 | 99(100) | 99(82) |
| NEU1 | 100 | 100(100) | 100(99) | 99 | 99(100) | 99(99) |
| NEUROD1 | 98 | 98(100) | 99(85) | 98 | 98(100) | 98(84) |
| NFIA | 98 | 98(100) | 99(84) | 95 | 95(100) | 95(89) |
| NFKBIZ | 97 | 98(100) | 98(98) | 96 | 96(99) | 97(93) |
| NHN1 | 97 | 97(100) | 99(75) | 94 | 94(99) | 95(69) |
| NKD1 | 99 | 99(100) | 99(98) | 97 | 98(100) | 98(98) |
| NLF1 | 100 | 99(100) | 100(95) | 100 | 100(100) | 100(92) |
| NMNAT3 | 99 | 99(100) | 99(97) | 98 | 98(100) | 98(96) |
| NOL6 | 94 | 95(99) | 98(61) | 91 | 92(94) | 93(62) |
| NOVA1 | 100 | 100(100) | 100(97) | 100 | 100(100) | 100(96) |
| NPR1 | 95 | 95(100) | 99(76) | 95 | 95(99) | 97(76) |
| NQO1 | 99 | 99(99) | 99(97) | 98 | 98(98) | 98(98) |
| NRAS | 100 | 100(100) | 100(100) | 100 | 100(100) | 100(100) |
| NSUN7 | 98 | 98(99) | 99(96) | 97 | 97(99) | 98(93) |
| NT5C2 | 99 | 99(100) | 99(98) | 97 | 97(100) | 97(97) |
| NUDT1 | 99 | 99(99) | 100(95) | 97 | 97(100) | 97(97) |
| NUDT5 | 98 | 98(99) | 100(87) | 98 | 98(100) | 99(88) |
| NUDT9 | 99 | 99(100) | 100(94) | 98 | 98(100) | 98(94) |
| NUFIP1 | 99 | 99(100) | 99(89) | 96 | 96(100) | 95(89) |
| NUMB | 100 | 100(100) | 100(98) | 98 | 98(100) | 98(98) |
| NYD-SP18 | 98 | 98(100) | 98(92) | 96 | 96(100) | 96(92) |
| OBP2B | 99 | 99(100) | 99(99) | 100 | 100(100) | 100(100) |
| ODC1 | 97 | 97(100) | 99(90) | 97 | 97(100) | 97(90) |
| OGG1 | 100 | 100(100) | 100(98) | 99 | 99(100) | 99(98) |
| OLFM1 | 98 | 98(100) | 98(90) | 95 | 95(100) | 95(87) |
| OPTC | 98 | 98(100) | 99(88) | 96 | 97(98) | 97(89) |
| OR10J1 | 97 | 97(99) | 99(88) | 93 | 94(96) | 95(78) |
| OR13A1 | 99 | 99(100) | 99(96) | 97 | 97(100) | 97(96) |
| OR2D3 | 96 | 96(98) | 99(65) | 93 | 93(96) | 94(63) |
| OR4D11 | 98 | 98(100) | 99(95) | 97 | 97(100) | 98(94) |
| OR51B4 | 94 | 95(96) | 99(64) | 85 | 87(92) | 89(60) |
| OR52H1 | 96 | 96(99) | 99(71) | 92 | 93(98) | 95(75) |
| OR56A4 | 99 | 99(100) | 99(99) | 97 | 97(100) | 98(96) |
| OR6C76 | 99 | 99(100) | 99(93) | 96 | 96(100) | 97(94) |
| OR8A1 | 95 | 95(99) | 98(44) | 92 | 93(99) | 96(47) |
| OSGIN2 | 99 | 99(100) | 100(91) | 98 | 98(100) | 98(90) |
| OXA1L | 97 | 97(99) | 99(83) | 96 | 97(98) | 97(83) |
| P2RX2 | 99 | 99(100) | 100(99) | 99 | 99(100) | 99(98) |
| PACS1 | 99 | 99(100) | 100(94) | 96 | 96(100) | 98(89) |
| PAK1 | 100 | 100(100) | 100(100) | 99 | 99(100) | 99(99) |
| PAK4 | 100 | 100(100) | 100(97) | 98 | 98(99) | 98(96) |
| PAQR5 | 97 | 97(98) | 99(82) | 95 | 95(99) | 97(74) |
| PAWR | 97 | 97(100) | 98(95) | 95 | 95(100) | 96(94) |
| PAX4 | 98 | 98(100) | 99(92) | 96 | 96(100) | 96(93) |
| PBK | 98 | 98(100) | 99(89) | 97 | 97(99) | 97(86) |
| PBX3 | 99 | 99(100) | 99(99) | 98 | 98(100) | 98(100) |
| PBX4 | 100 | 100(100) | 100(99) | 99 | 99(100) | 99(99) |
| PCBD1 | 100 | 100(100) | 100(99) | 100 | 100(100) | 100(100) |
| PCDH10 | 98 | 98(100) | 98(93) | 97 | 97(100) | 98(90) |
| PCDHGC4 | 99 | 99(100) | 100(92) | 99 | 99(100) | 99(94) |
| PCTK3 | 96 | 96(99) | 98(75) | 91 | 91(99) | 92(71) |
| PDE8B | 98 | 98(100) | 99(93) | 94 | 94(98) | 96(84) |
| PDGFB | 98 | 98(100) | 100(92) | 98 | 98(99) | 99(89) |
| PDSS1 | 99 | 99(100) | 99(96) | 99 | 99(100) | 99(96) |
| PDZD8 | 98 | 98(100) | 99(83) | 92 | 92(99) | 94(81) |
| PF4 | 98 | 98(100) | 100(93) | 98 | 98(100) | 99(96) |
| PGBD1 | 96 | 96(100) | 99(77) | 95 | 95(100) | 95(83) |
| PHF15 | 97 | 97(100) | 99(82) | 97 | 97(100) | 98(89) |
| PHLDA3 | 99 | 99(100) | 99(98) | 99 | 99(100) | 99(99) |
| PI4K2B | 99 | 99(100) | 99(94) | 98 | 98(99) | 98(92) |
| PIAS2 | 98 | 98(100) | 100(90) | 98 | 98(100) | 99(89) |
| PIGL | 99 | 99(100) | 99(91) | 99 | 99(100) | 99(92) |
| PIGP | 99 | 99(99) | 99(95) | 99 | 99(100) | 99(94) |
| PIGS | 99 | 99(99) | 100(92) | 98 | 98(100) | 98(92) |
| PIK3R3 | 99 | 99(100) | 99(94) | 96 | 96(100) | 97(93) |
| PIP5K2B | 99 | 99(100) | 99(99) | 97 | 97(100) | 99(97) |
| PLAT | 95 | 95(99) | 97(74) | 92 | 92(98) | 92(72) |
| PLCD1 | 97 | 97(100) | 98(86) | 92 | 93(96) | 94(80) |
| PMM2 | 99 | 99(100) | 100(96) | 98 | 98(100) | 98(96) |
| PMP22CD | 97 | 97(100) | 99(79) | 95 | 95(98) | 97(75) |
| PODXL2 | 98 | 98(100) | 99(94) | 94 | 94(100) | 95(92) |
| PON2 | 99 | 99(100) | 99(97) | 97 | 97(100) | 97(95) |
| PPAT | 96 | 96(100) | 99(71) | 95 | 95(100) | 99(71) |
| PPIG | 98 | 98(100) | 99(84) | 92 | 92(96) | 92(77) |
| PPM1J | 97 | 97(99) | 100(77) | 89 | 90(94) | 93(74) |
| PPP1R13L | 100 | 100(100) | 100(97) | 99 | 99(100) | 99(96) |
| PPP1R16A | 100 | 100(100) | 100(100) | 100 | 100(100) | 100(100) |
| PPP2R1B | 97 | 97(100) | 99(75) | 96 | 96(99) | 98(70) |
| PPP2R5C | 97 | 97(100) | 99(90) | 94 | 95(99) | 96(87) |
| PPP2R5D | 100 | 100(100) | 100(100) | 100 | 100(100) | 100(100) |
| PQLC1 | 99 | 99(100) | 99(96) | 96 | 96(100) | 96(93) |
| PRG3 | 97 | 97(100) | 98(72) | 95 | 95(100) | 97(71) |
| PRMT5 | 99 | 99(100) | 100(89) | 96 | 97(100) | 98(86) |
| PRPF40B | 98 | 98(100) | 99(87) | 98 | 98(100) | 98(87) |
| PSAT1 | 98 | 98(100) | 99(88) | 97 | 97(99) | 98(89) |
| PSEN1 | 99 | 99(100) | 100(92) | 99 | 99(100) | 100(89) |
| PSMB4 | 98 | 98(100) | 99(93) | 97 | 97(100) | 98(91) |
| PSMB8 | 99 | 99(100) | 99(95) | 96 | 96(98) | 97(93) |
| PSMD2 | 99 | 99(100) | 100(98) | 99 | 99(100) | 99(98) |
| PSMD7 | 100 | 100(100) | 100(100) | 100 | 100(100) | 100(100) |
| PSME2 | 97 | 97(99) | 99(87) | 96 | 96(100) | 97(87) |
| PSRC1 | 97 | 97(100) | 99(82) | 89 | 89(95) | 92(73) |
| PTGER3 | 100 | 100(100) | 100(94) | 96 | 96(100) | 97(93) |
| PTGFR | 98 | 98(98) | 100(86) | 95 | 97(93) | 97(80) |
| PTPN9 | 100 | 100(99) | 100(98) | 100 | 100(100) | 100(100) |
| PYGM | 98 | 98(100) | 99(98) | 97 | 97(100) | 97(97) |
| QPCT | 98 | 98(100) | 98(91) | 97 | 97(99) | 97(85) |
| R3HDM1 | 98 | 98(100) | 99(92) | 96 | 96(100) | 96(91) |
| RAB36 | 98 | 98(100) | 98(99) | 97 | 97(100) | 97(98) |
| RAB38 | 97 | 97(100) | 98(96) | 98 | 98(100) | 98(93) |
| RAB40B | 99 | 99(100) | 99(100) | 98 | 98(100) | 98(99) |
| RAD51AP1 | 99 | 99(100) | 100(92) | 98 | 98(100) | 100(88) |
| RAD54B | 94 | 94(100) | 99(63) | 88 | 88(97) | 95(60) |
| RALY | 99 | 99(100) | 99(100) | 100 | 100(100) | 100(99) |
| RAN | 99 | 99(99) | 99(95) | 99 | 99(98) | 99(92) |
| RARB | 99 | 99(99) | 99(90) | 96 | 96(100) | 97(89) |
| RBPSUH | 96 | 97(98) | 98(83) | 90 | 90(98) | 92(78) |
| RCL1 | 100 | 100(100) | 99(95) | 97 | 97(100) | 97(97) |
| RCN2 | 99 | 99(98) | 99(91) | 99 | 99(99) | 99(91) |
| RDH11 | 100 | 100(100) | 100(97) | 98 | 98(100) | 98(100) |
| RDH12 | 99 | 99(100) | 99(94) | 98 | 98(100) | 98(95) |
| RECQL5 | 98 | 98(100) | 99(91) | 97 | 97(99) | 99(85) |
| REXO2 | 99 | 99(99) | 100(93) | 98 | 98(100) | 99(92) |
| RFXANK | 98 | 98(100) | 99(81) | 95 | 95(99) | 98(66) |
| RGS7 | 99 | 99(98) | 99(95) | 98 | 98(99) | 98(96) |
| RHOH | 100 | 100(100) | 100(100) | 99 | 99(100) | 99(100) |
| RHOT1 | 99 | 99(100) | 99(95) | 98 | 98(100) | 98(94) |
| RIC8A | 99 | 99(100) | 99(93) | 95 | 95(100) | 95(95) |
| RIOK1 | 98 | 98(100) | 99(74) | 95 | 95(99) | 95(74) |
| RIPK1 | 99 | 99(100) | 99(93) | 97 | 97(100) | 97(88) |
| RIPK3 | 98 | 98(99) | 99(87) | 98 | 98(100) | 98(87) |
| RNASE6 | 98 | 98(100) | 99(86) | 96 | 96(100) | 97(86) |
| RNF113B | 98 | 98(99) | 99(92) | 93 | 93(99) | 96(81) |
| RNF122 | 98 | 98(100) | 99(91) | 97 | 97(98) | 98(91) |
| RNF150 | 97 | 97(100) | 98(82) | 94 | 94(100) | 94(83) |
| RNF25 | 99 | 99(100) | 99(99) | 96 | 96(100) | 96(99) |
| RNF44 | 100 | 100(100) | 100(96) | 99 | 99(100) | 99(98) |
| RNMT | 99 | 99(100) | 99(94) | 96 | 96(100) | 97(89) |
| RPL27A | 100 | 100(100) | 100(98) | 99 | 99(100) | 99(98) |
| RPS24 | 100 | 100(100) | 100(97) | 99 | 99(100) | 99(97) |
| RRAS | 99 | 99(100) | 100(95) | 97 | 97(100) | 97(96) |
| RUNX2 | 99 | 99(100) | 99(97) | 98 | 98(99) | 99(95) |
| SAMD14 | 100 | 100(100) | 100(97) | 99 | 99(100) | 99(96) |
| SART3 | 97 | 97(100) | 100(83) | 95 | 95(99) | 98(78) |
| SCGB2A2 | 99 | 99(100) | 99(99) | 99 | 99(100) | 99(99) |
| SDF2L1 | 99 | 99(100) | 99(93) | 97 | 97(100) | 98(92) |
| SEC11C | 100 | 100(99) | 100(98) | 98 | 98(100) | 99(97) |
| SEC13 | 99 | 99(100) | 100(97) | 99 | 99(100) | 99(95) |
| SEC14L3 | 97 | 97(99) | 99(83) | 91 | 92(96) | 92(68) |
| SERAC1 | 98 | 98(100) | 99(90) | 93 | 93(99) | 94(89) |
| SERPINA11 | 98 | 98(100) | 99(85) | 94 | 94(100) | 94(87) |
| SETD2 | 96 | 96(99) | 98(67) | 94 | 95(98) | 95(66) |
| SETD8 | 100 | 100(100) | 100(99) | 100 | 100(100) | 100(99) |
| SF3B14 | 100 | 100(100) | 100(99) | 100 | 100(100) | 100(98) |
| SFRS1 | 100 | 100(100) | 100(99) | 100 | 100(100) | 100(99) |
| SGCB | 100 | 100(100) | 100(98) | 99 | 99(100) | 100(99) |
| SGK3 | 99 | 99(100) | 99(98) | 98 | 98(100) | 98(97) |
| SGPL1 | 99 | 99(100) | 99(95) | 99 | 99(100) | 99(96) |
| SIAH1 | 100 | 100(100) | 100(100) | 100 | 100(100) | 100(100) |
| SIRT1 | 98 | 98(100) | 99(93) | 96 | 96(100) | 96(91) |
| SKAP1 | 99 | 99(100) | 99(96) | 96 | 96(100) | 98(94) |
| SLAMF8 | 97 | 97(100) | 99(79) | 89 | 89(99) | 92(76) |
| SLC12A2 | 97 | 97(100) | 99(88) | 97 | 97(100) | 98(81) |
| SLC16A10 | 99 | 99(100) | 99(96) | 99 | 99(100) | 99(94) |
| SLC17A3 | 99 | 99(100) | 99(97) | 95 | 95(99) | 96(96) |
| SLC25A31 | 99 | 99(100) | 100(95) | 96 | 96(100) | 97(94) |
| SLC26A2 | 98 | 99(98) | 99(94) | 95 | 96(98) | 96(93) |
| SLC30A1 | 99 | 99(100) | 99(96) | 99 | 99(100) | 99(96) |
| SLC30A3 | 100 | 100(99) | 100(98) | 99 | 99(100) | 99(97) |
| SLC34A2 | 97 | 98(98) | 100(78) | 88 | 89(96) | 92(70) |
| SLC35B1 | 99 | 99(100) | 100(95) | 99 | 99(100) | 100(94) |
| SLC3A2 | 97 | 97(100) | 98(90) | 96 | 97(99) | 97(87) |
| SLC43A1 | 99 | 99(100) | 99(96) | 97 | 98(99) | 98(94) |
| SLC44A2 | 98 | 98(100) | 99(99) | 98 | 98(100) | 99(97) |
| SLC4A1AP | 97 | 97(100) | 97(91) | 95 | 95(99) | 96(87) |
| SLCO1A2 | 97 | 97(100) | 99(87) | 95 | 96(100) | 97(85) |
| SMC5 | 98 | 98(100) | 99(86) | 94 | 94(99) | 98(79) |
| SNX5 | 99 | 99(100) | 99(97) | 97 | 97(100) | 98(97) |
| SOCS4 | 99 | 99(100) | 100(92) | 98 | 98(100) | 99(91) |
| SPINK2 | 100 | 100(100) | 100(100) | 99 | 99(100) | 99(99) |
| SPP2 | 99 | 99(100) | 100(90) | 96 | 96(100) | 97(93) |
| SPRR2G | 100 | 100(100) | 100(99) | 100 | 100(100) | 100(98) |
| SPTLC2 | 97 | 97(99) | 99(87) | 96 | 97(96) | 98(84) |
| SRGAP1 | 97 | 98(99) | 98(89) | 95 | 96(96) | 97(85) |
| SRPR | 97 | 97(100) | 99(89) | 96 | 96(100) | 96(92) |
| SS18L1 | 99 | 99(99) | 99(96) | 96 | 96(100) | 97(96) |
| ST3GAL3 | 99 | 99(100) | 99(96) | 98 | 98(100) | 98(96) |
| ST3GAL6 | 99 | 99(100) | 100(97) | 99 | 99(100) | 99(99) |
| ST6GALNAC3 | 98 | 98(100) | 98(94) | 98 | 98(100) | 98(89) |
| ST7L | 97 | 97(100) | 98(81) | 93 | 93(100) | 93(76) |
| STK38L | 100 | 100(100) | 100(98) | 98 | 98(100) | 99(98) |
| STOM | 100 | 100(100) | 100(100) | 99 | 99(100) | 99(100) |
| STOML1 | 97 | 97(100) | 99(81) | 96 | 96(100) | 97(81) |
| STX16 | 98 | 98(100) | 100(87) | 91 | 92(98) | 93(72) |
| STXBP3 | 97 | 97(99) | 99(84) | 96 | 97(99) | 98(85) |
| STXBP4 | 99 | 99(100) | 99(96) | 97 | 97(100) | 97(97) |
| SUNC1 | 99 | 99(100) | 100(93) | 98 | 98(100) | 99(88) |
| SUZ12 | 100 | 100(100) | 100(99) | 99 | 99(100) | 100(99) |
| SYNGR1 | 99 | 99(99) | 100(98) | 99 | 99(99) | 100(98) |
| SYNGR2 | 99 | 99(99) | 100(85) | 96 | 96(98) | 97(83) |
| SYNJ1 | 98 | 98(100) | 99(94) | 95 | 96(98) | 96(90) |
| SYTL2 | 97 | 97(99) | 99(77) | 92 | 94(93) | 94(71) |
| TAF11 | 99 | 99(100) | 99(100) | 98 | 98(100) | 98(98) |
| TARS2 | 97 | 97(100) | 99(84) | 96 | 96(100) | 98(84) |
| TEX14 | 96 | 97(99) | 99(75) | 87 | 88(96) | 91(73) |
| THOC6 | 99 | 99(100) | 99(100) | 98 | 98(100) | 98(99) |
| THRB | 100 | 100(100) | 100(97) | 98 | 98(100) | 98(96) |
| TIA1 | 98 | 98(100) | 99(90) | 96 | 96(100) | 97(87) |
| TIAF1 | 98 | 98(100) | 100(83) | 98 | 98(100) | 99(80) |
| TIMM8B | 100 | 100(100) | 100(99) | 100 | 100(100) | 100(100) |
| TIPRL | 99 | 99(100) | 100(97) | 100 | 100(100) | 100(97) |
| TKTL2 | 97 | 97(99) | 99(84) | 94 | 94(98) | 96(83) |
| TLL1 | 97 | 98(98) | 100(78) | 79 | 83(81) | 86(50) |
| TMCC1 | 100 | 100(100) | 100(93) | 95 | 95(100) | 95(96) |
| TMEM101 | 99 | 99(100) | 100(97) | 98 | 98(100) | 99(96) |
| TMEM106C | 98 | 98(100) | 98(93) | 97 | 97(99) | 97(89) |
| TMEM133 | 99 | 99(100) | 99(96) | 98 | 98(99) | 98(95) |
| TMEM177 | 98 | 98(100) | 99(87) | 94 | 95(98) | 96(81) |
| TMEM182 | 100 | 100(99) | 100(98) | 99 | 99(99) | 99(98) |
| TMEM63A | 92 | 95(90) | 99(57) | 86 | 88(92) | 92(54) |
| TMEM67 | 99 | 99(100) | 99(96) | 98 | 98(100) | 98(96) |
| TMOD2 | 100 | 100(100) | 100(100) | 100 | 100(100) | 100(99) |
| TMPRSS11D | 100 | 100(100) | 100(98) | 99 | 99(100) | 99(99) |
| TOP3B | 97 | 97(99) | 99(88) | 95 | 95(99) | 97(86) |
| TOR1A | 98 | 98(100) | 99(91) | 98 | 98(100) | 98(93) |
| TPM3 | 100 | 100(100) | 100(100) | 100 | 100(100) | 100(100) |
| TPM4 | 98 | 98(100) | 100(84) | 92 | 92(98) | 95(78) |
| TPSD1 | 96 | 96(99) | 97(84) | 97 | 97(100) | 98(86) |
| TRAFD1 | 99 | 99(100) | 99(97) | 97 | 97(100) | 97(98) |
| TRAPPC2L | 99 | 99(100) | 99(95) | 95 | 95(100) | 97(93) |
| TRIM2 | 98 | 98(99) | 99(83) | 94 | 95(98) | 96(81) |
| TRIM3 | 98 | 98(100) | 99(86) | 98 | 98(99) | 98(80) |
| TRIM33 | 99 | 99(100) | 99(99) | 97 | 97(98) | 97(96) |
| TRIM55 | 100 | 100(100) | 100(99) | 100 | 100(100) | 100(100) |
| TRIM62 | 98 | 98(100) | 99(88) | 96 | 96(100) | 96(88) |
| TRMT5 | 99 | 99(100) | 99(89) | 96 | 96(100) | 98(88) |
| TRPC4 | 96 | 96(97) | 100(74) | 94 | 95(94) | 97(68) |
| TSPAN3 | 99 | 99(100) | 99(95) | 98 | 98(99) | 98(94) |
| TTC26 | 99 | 99(100) | 99(89) | 97 | 97(100) | 99(84) |
| TTLL11 | 99 | 99(100) | 100(94) | 97 | 97(100) | 98(93) |
| TTLL13 | 98 | 98(100) | 98(92) | 96 | 96(100) | 97(88) |
| TWF2 | 98 | 98(100) | 100(84) | 94 | 94(99) | 98(73) |
| TXNL1 | 99 | 99(100) | 100(93) | 98 | 98(100) | 99(91) |
| U2AF1 | 99 | 99(100) | 100(93) | 97 | 98(100) | 99(94) |
| UAP1 | 97 | 97(100) | 100(76) | 96 | 96(99) | 98(75) |
| UBE2V1 | 100 | 100(100) | 100(98) | 99 | 99(100) | 99(98) |
| UGT1A6 | 99 | 99(100) | 99(94) | 96 | 96(100) | 96(96) |
| ULBP1 | 99 | 99(100) | 100(95) | 98 | 98(99) | 99(91) |
| UNC50 | 100 | 100(100) | 100(99) | 100 | 100(100) | 100(100) |
| UNQ9438 | 100 | 100(100) | 100(98) | 100 | 100(100) | 100(98) |
| UPP1 | 99 | 99(100) | 99(97) | 98 | 98(100) | 99(97) |
| VAMP1 | 99 | 99(100) | 99(98) | 98 | 98(100) | 98(98) |
| VAMP3 | 100 | 100(100) | 100(100) | 99 | 99(100) | 99(100) |
| VGF | 99 | 99(100) | 100(96) | 99 | 99(100) | 99(93) |
| VIM | 100 | 100(100) | 100(100) | 99 | 99(100) | 99(100) |
| VLDLR | 98 | 98(100) | 98(94) | 93 | 94(98) | 96(87) |
| VN1R1 | 98 | 98(100) | 99(90) | 98 | 98(98) | 98(85) |
| VNN3 | 96 | 96(100) | 99(65) | 94 | 94(98) | 97(61) |
| VTCN1 | 99 | 99(99) | 100(89) | 96 | 96(100) | 99(84) |
| VTI1B | 99 | 99(100) | 99(91) | 97 | 97(100) | 99(89) |
| WDR8 | 98 | 98(100) | 99(83) | 94 | 94(100) | 94(87) |
| WFDC3 | 99 | 99(100) | 100(94) | 96 | 97(99) | 99(93) |
| WFDC5 | 98 | 98(100) | 99(90) | 96 | 96(100) | 97(89) |
| WNT16 | 95 | 95(100) | 96(79) | 93 | 93(98) | 94(76) |
| XRCC5 | 98 | 98(100) | 99(93) | 97 | 97(100) | 97(92) |
| YOD1 | 98 | 98(99) | 99(88) | 96 | 96(98) | 98(89) |
| ZBTB16 | 99 | 99(100) | 99(97) | 96 | 96(100) | 96(98) |
| ZBTB26 | 100 | 100(100) | 100(100) | 100 | 100(100) | 100(100) |
| ZDHHC6 | 98 | 98(100) | 98(86) | 97 | 97(99) | 98(83) |
| ZFP2 | 98 | 98(99) | 99(88) | 92 | 92(100) | 94(86) |
| ZFYVE21 | 98 | 98(100) | 100(82) | 95 | 95(100) | 97(76) |
| ZKSCAN5 | 98 | 99(97) | 99(96) | 98 | 99(97) | 99(97) |
| ZMPSTE24 | 99 | 99(100) | 100(95) | 97 | 97(100) | 97(94) |
| ZMYM5 | 99 | 99(100) | 99(94) | 99 | 99(100) | 99(95) |
| ZNF213 | 98 | 98(99) | 99(87) | 97 | 97(99) | 97(83) |
| ZNF26 | 98 | 98(100) | 98(90) | 95 | 96(98) | 97(85) |
| ZNF264 | 97 | 97(100) | 99(83) | 94 | 95(99) | 95(81) |
| ZNF32 | 100 | 100(100) | 100(96) | 99 | 99(100) | 100(95) |
| ZNF329 | 98 | 98(100) | 99(92) | 98 | 98(100) | 98(93) |
| ZNF396 | 99 | 99(100) | 100(99) | 99 | 99(100) | 99(99) |
| ZNF474 | 88 | 88(93) | 95(22) | 80 | 82(88) | 84(20) |
| ZNF521 | 95 | 97(98) | 100(63) | 84 | 84(86) | 82(44) |
| ZNF555 | 98 | 98(100) | 100(84) | 95 | 95(99) | 98(80) |
| ZNF571 | 98 | 98(100) | 98(92) | 92 | 92(100) | 94(91) |
| ZNF576 | 99 | 99(100) | 99(90) | 96 | 96(100) | 97(90) |
| ZNF583 | 99 | 99(100) | 99(93) | 98 | 98(99) | 98(91) |
| ZNF616 | 94 | 95(97) | 98(58) | 88 | 89(96) | 92(54) |
| ZNF625 | 99 | 99(100) | 99(100) | 97 | 97(100) | 97(98) |
| ZNF642 | 99 | 99(100) | 100(93) | 97 | 97(100) | 98(91) |
| ZNF689 | 99 | 100(99) | 100(95) | 98 | 98(100) | 98(95) |
| ZNHIT4 | 100 | 100(100) | 100(100) | 99 | 99(100) | 99(100) |
| ZPBP2 | 92 | 92(100) | 98(44) | 86 | 86(100) | 87(43) |
| ZSCAN2 | 100 | 100(100) | 100(100) | 100 | 100(100) | 100(100) |
| ZSWIM1 | 98 | 98(100) | 100(87) | 96 | 96(98) | 97(86) |
